# Metabarcoding of Hepatitis E virus genotype 3 and Norovirus GII from wastewater samples in England using nanopore sequencing

**DOI:** 10.1007/s12560-023-09569-w

**Published:** 2023-11-01

**Authors:** Samantha Treagus, James Lowther, Ben Longdon, William Gaze, Craig Baker-Austin, David Ryder, Frederico M. Batista

**Affiliations:** 1 Centre for Environment, Fisheries and Aquaculture Science, Weymouth, United Kingdom; 2 Centre for Ecology and Conservation, Faculty of Environment, Science and Economy, University of Exeter, Penryn campus, Cornwall, United Kingdom; 3 Faculty of Health and Life Sciences, University of Exeter Medical School, Penryn campus, Cornwall, United Kingdom

**Keywords:** Hepatitis E virus, Norovirus, nanopore sequencing, metabarcoding, environmental transmission, wastewater-based epidemiology

## Abstract

Norovirus is one of the largest causes of gastroenteritis worldwide, and Hepatitis E virus (HEV) is an emerging pathogen that has become the most dominant cause of acute viral hepatitis in recent years. The presence of norovirus and HEV has been reported within wastewater in many countries previously. Here we used amplicon deep sequencing (metabarcoding) to identify norovirus and HEV strains in wastewater samples from England collected in 2019 and 2020. For HEV, we sequenced a fragment of the RNA-dependent RNA polymerase (RdRp) gene targeting genotype 3 strains. For norovirus, we sequenced the 5’ portion of the major capsid protein gene (VP1) of genogroup II strains. Sequencing of the wastewater samples revealed eight different genotypes of norovirus GII (GII.2, GII.3, GII.4, GII.6, GII.7, GII.9, GII.13 and GII.17). Genotypes GII.3 and GII.4 were the most commonly found. The HEV metabarcoding assay was able to identify HEV genotype 3 strains in some samples with a very low viral concentration determined by RT-qPCR. Analysis showed that most HEV strains found in influent wastewater were typed as G3c and G3e and were likely to have originated from humans or swine. However, the small size of the HEV nested PCR amplicon could cause issues with typing, and so this method is more appropriate for samples with high CTs where methods targeting longer genomic regions are unlikely to be successful. This is the first report of HEV RNA in wastewater in England. This study demonstrates the utility of wastewater sequencing and the need for wider surveillance of norovirus and HEV within host species and environments.

## Introduction

Norovirus and hepatitis E virus (HEV) are enteric viruses that are often overlooked in clinical research due to their relatively non-severe nature, the short symptomatic period for norovirus, and the relatively low prevalence of HEV in most countries (Htet, Althaf and Karim, 2018; Robilotti, Deresinski and Pinsky, 2015; Lhomme *et al*., 2020; Public Health England, 2019). However, norovirus causes a significant annual healthcare burden of 685 million cases and $60 billion globally (Centers for Disease Control and Prevention, 2021), and HEV genotypes 1 and 2 alone were estimated to cause 20 million cases annually in 2002 (World Health Organization, 2021). The most recent data on cases available is from a study of 30 European countries, which showed that confirmed HEV cases increased from 514 to 5,617 cases between 2005 and 2015 (Aspinall *et al*., 2017). Both viruses can also have severe and fatal outcomes in immunocompromised people. Genome sequencing has become an invaluable tool for understanding the epidemiology and evolution of viruses. It can be used to track the evolution of new variants and to understand the phylogeography and spread of a virus (Medema *et al*., 2020; Martin *et al*., 2020; Agrawal et al., 2021). Due to the widespread adoption and success of sequencing viruses such as severe acute respiratory syndrome coronavirus 2 (SARS-CoV-2) from wastewater, researchers are looking to monitor other viruses to improve knowledge of their abundance within communities.

Norovirus and HEV spread through faeco-oral and foodborne routes of transmission. Outbreaks of norovirus are usually spread through person-to-person transmission but can also be spread through consumption of contaminated foods or through fomites (Meghnath *et al*., 2019; Prato *et al*., 2004). Genome sequencing has been used for identifying genogroups, genotypes and strains of norovirus that cause norovirus outbreaks and to determine the source of the infection. A large study by (Verhoef *et al*., 2011) used sequencing data to identify the global sources and relationships between norovirus outbreaks and conservatively estimated that 7% of norovirus outbreaks had an international geographical distribution. These findings contrasted with previous estimates of 0.4% through standard epidemiological investigations (Robilotti et al., 2015; Lu *et al*., 2016; Sakon *et al*., 2018).

HEV, on the other hand, usually spreads through the consumption of undercooked meat, particularly pork (VanderWaal and Deen, 2018; Guillois *et al*., 2015). A study on the prevalence of HEV in UK pigs identified a seroprevalence of 92.8%, and HEV RNA presence in 21% of the animals analysed (Grierson *et al*., 2015), suggesting that consumption of pork is a significant risk factor in the UK. However, many possible host animal species for HEV have been identified (Kenney, 2019). HEV has been detected in vegetarians in the Netherlands (Slot *et al*., 2017), suggesting there may be routes of infection other than consumption of animal products. As such, e-tracing sources of outbreaks using sequencing may provide insights into other transmission routes of HEV, in particular from environmental sources.

Viruses in food and environmental samples are often present at very low abundance, which can make detection and genotyping by nucleotide sequencing difficult. Consequently, PCR-based methods are often used to amplify a specific genomic region for sequencing. Most studies on food and environmental samples to date have used PCR-based methods followed by Sanger sequencing (Rivadulla *et al*., 2019; Pallerla *et al*., 2021). Unlike high throughput sequencing (HTS) techniques, Sanger sequencing is less likely to pick up rare variants within a sample or may lead to unresolved bases at certain divergent loci where samples contain multiple divergent strains at similar concentrations (Mancini *et al*., 2019; Gao *et al*., 2016). HTS is becoming more commonly used to identify norovirus in different food matrices, for example in food products such as strawberries (Bartsch *et al*., 2018), and shellfish (Ollivier *et al*., 2022). However, application of HTS to identify norovirus and HEV in wastewater samples in the UK has not yet been performed to the best of our knowledge.

The aim of this study was to develop a HTS metabarcoding approach using Oxford Nanopore Technologies (ONT) to identify and characterise norovirus and HEV present in wastewater samples at low levels. ONT was chosen since the cost of the sequencing devices is low, and it is a scalable sequencing platform, allowing the analyses of a variable number of samples on the same flow cell.

## Materials and Methods

### Wastewater samples

We collected 140 paired grab samples, comprising of 70 influent and 70 effluent samples, from seven wastewater treatment plants (WWTPs) in Southern England between October 2019 and February 2020. Influent samples consisted of a litre of coarsely screened influent, and effluent samples were one litre of final effluent. Ten pairs of influent and effluent samples were collected from each WWTP over the collection period. Samples were collected weekly or fortnightly during this period. Table 1 shows the population served and treatment level for each WWTP.

### Wastewater virus extraction

Samples were concentrated using a modified ultracentrifugation method adapted from Puig *et al*., (1994). Briefly, 40 ml of wastewater sample was mixed, then split equally into two ultracentrifuge bottles. Mengo virus (10 μl) was then added to both bottles to act as a recovery control. Sample bottles were spun at 152,000 x g at 4°C for one hour and the supernatant was discarded. The pellet from one of the sample bottles was resuspended in 2 ml of 0.25 M glycine buffer (pH 9.5; glycine powder from Sigma-Aldrich, St. Louis, MO, USA). This suspension was then added to the pellet from the second sample bottle and the second pellet resuspended with the first. The sample was then put on ice for 20 min before adding 2ml of cold (4°C) phosphate buffered saline (PBS tablets, VWR, Radnor, PA, USA). Samples were centrifuged at 6,090 x g for 20 min at 4°C. The supernatant was transferred to clean bottles, and 18 ml of cold PBS added before a final spin at 152,000 x g at 4°C for one hour. The pellet from this final spin was resuspended in 1ml of cold PBS before it was used in subsequent RNA extraction.

### RNA extraction

Five hundred microlitres of concentrated wastewater pellet was added to 2 ml of lysis buffer (Biomerieux, Durham, NC, USA) and incubated at room temperature for 10 min. Total RNA was then extracted using a viral RNA extraction method developed for food samples, described in ISO 15216-1:2017 and Lowther *et al*. (2012). Following the lysis buffer incubation, 50 μl of magnetic silica beads from the NucliSENS Magnetic Extraction Reagents kit (Biomerieux) were added, and the mixture was left to incubate for ten minutes at room temperature. The sample was then centrifuged at 1500 x g for 2 minutes and the supernatant discarded. The silica beads remaining were washed in buffers whilst held in a NucliSENS Minimag (Biomerieux). Wash buffer 1 was applied and removed using an aspirator after 30 seconds of wash spinning within the Minimag; and this step was repeated. Wash buffer 2 was then applied in the same way and repeated. Wash buffer 3 was applied for 15 seconds of wash spinning and removed, and finally 100 μl of elution buffer was added to resuspend the magnetic beads. The elution mix was incubated on a thermoshaker at 60°C and 1400 rpm for five minutes, before applying to a magnet to separate the buffer (containing extracted RNA) from the silica beads. The 100 μl of RNA extract was then transferred to a clean tube for use in subsequent qRT-PCR. A reference extraction was performed alongside the samples, consisting of 10 μl of mengo virus (same batch as used in the ultracentrifugation) in 500 μl of molecular grade water; and a negative extraction control of 500 μl of molecular grade water was also included during each extraction. The RNA extract was frozen at -80°C prior to testing.

### Detection and Quantification

RNA extracts were tested for the presence of HEV and norovirus GII using RT-qPCR. The detection of norovirus was carried out as described in the international standard for quantification of viruses in foods ISO 15216-1:2017 (International Organization for Standardization, 2017). The details for these assays and the HEV assay are shown in Table 2. The RT-qPCR assays for HEV and norovirus were prepared using RNA UltraSense™ One-Step Quantitative RT-PCR System (superscript III, Invitrogen, ThermoFisher Scientific Waltham, MA, USA) and thermal cycling and monitoring of amplicon formation was carried out using a QuantStudio 3 Real-Time PCR System. For the HEV and mengo virus RT-qPCR assays the final primer concentrations were 0.625 μM for the forward primer. The final concentration of the reverse primer for the HEV and mengo virus assays was 1.125 μM. For the norovirus GII forward primer the final concentration was 1 μM, and 1.8 μM for the norovirus GII reverse primer.

Cycling conditions for all PCRs were 55°C for 60 min for the reverse transcription, followed by 95°C for 5 min, then 45 cycles of 95°C for 15 sec, 60°C for 1 min and 65°C for 1 min. Synthetic DNA controls for production of standard curves for quantification were prepared following ISO 15216-1:2017 guidelines for norovirus GII, and a similar approach was used for HEV controls. Standard curves conformed to an r^2^ value ≥0.99 and a slope of between -3.1 and -3.6. Testing of norovirus GII and HEV followed the approach of Lowther *et al*. (2012) and ISO 15216-1:2017 for controls (International Organization for Standardization, 2017). Virus concentrations in wastewater (copies/ml) were calculated from copies/μl in the sample RNA using conversion factors based on the volumes tested and concentration factors applied.

### Norovirus GII and HEV samples selected for sequencing

Forty-two (21 influent and 21 effluent) wastewater samples with norovirus GII CT values between 27 and 39 were selected for norovirus sequencing analysis. These were composed of three influent and three effluent samples with the lowest CT values from each of the seven WWTPs. Separately, 42 wastewater samples (31 influent and 11 effluent samples) that yielded CT values for HEV between 34 and 44 were selected for HEV sequencing analysis. This constituted all of the samples which tested positive for HEV by RT-qPCR out of the 140 samples analysed. The sample sets for norovirus and HEV sequencing were therefore different although some samples were selected for both sets.

### HEV semi-nested PCR primer design

Due to high HEV nucleotide diversity, primers were designed targeting genotype 3 (G3) only as G3 causes the majority of cases in the UK (Oeser *et al*., 2019). The G3 reference sequences used in this study were downloaded from the National Center for Biotechnology Information (NCBI) and aligned using Clustal Omega (Madeira *et al*., 2022). The alignment of the G3 genomes can be found in Online Resource 1 (DOI: 10.6084/m9.figshare.21900873). Possible primer sequences were identified manually by identifying conserved regions by eye, before testing sequences from these regions for self-complementarity and melting temperature suitability using the Multiple Primer Analyzer from ThermoFisher Scientific (ThermoFisher Scientific, NA) and Oligo Calc from Northwestern University (Kibbe, 2007). Primers were selected if they had a melting temperature of 56-65°C, length of 17-23 bases, GC content between 30-60%, and a maximum of 3 degenerate bases. Once primer candidates had been found, primer pairs were identified by their melting temperature similarity, the ability to be used in a semi-nested PCR assay, the size of the amplicons and lack of primer complementarity. The primer set selected for the semi-nested PCR targeted the RNA-dependent RNA polymerase (RdRp). This gene was chosen since it was suitable for use according to the criteria described above and has been used previously for HEV typing (Lin *et al*., 2015). The length of the first-round amplicon was 258 bp and the length of target sequence in the semi-nested amplicon (i.e. not including additional primer adapter sequences added by tagging onto the primer sequences) was 254 bp.

### cDNA synthesis and semi-nested PCR

Synthesis of cDNA utilised the Invitrogen SuperScript™ IV First-Strand Synthesis System (Invitrogen, ThermoFisher Scientific Waltham, MA, USA) and random hexamers (final concentration 2.5 μM), per the manufacturers protocol (template volume 10 μl, reaction volume 20 μl). An Eppendorf Mastercycler Nexus was used for cDNA synthesis as well as for the first and second rounds of the semi-nested PCRs (nPCR). The primers for the norovirus GII nPCR were described previously; and target the extreme 3’ end of the RdRp polymerase gene (ORF1) and the 5’ portion of the VP1 major capsid protein gene (ORF2). The primers used in the second round for both norovirus GII and HEV assays were modified with 5’ adapter sequences to allow addition of barcode sequences to enable multiplex sequencing. The primer sequences and reaction conditions are detailed in Table 3. The final concentration of both forward and reverse primers were 0.4 μM.

Amplification conditions for HEV G3 and norovirus GII PCRs were the same for the first and second rounds, and consisted of 95°C for 1 min, followed by 40 cycles of 95°C for 30 sec, 50°C for 30 sec, and 72°C for 30 sec. There was a final extension step of 72°C for 7 min. A negative control was utilised in all PCR reactions (water in place of sample cDNA). Negative controls were sequenced alongside samples providing positive nPCR results. A positive control was used for the first and second round PCRs for the norovirus GII assay, composed of cDNA synthesised from a norovirus GII LENTICULE (RMNOROG2, UK Health Security Agency). For G3 HEV, cDNA synthesized from RNA from cell culture was used as a positive control (RNA kindly donated by Eva Trojnar and Reimar Johne of Bundesintitut für Risikobewertung, Berlin, Germany). Positive controls were not sequenced.

The nPCR amplicons were visualised using 2% agarose (Scientific Laboratory Supplies) gel electrophoresis with Gel Red (10,000X in water, BIOTIUM) nucleic acid gel stain. PCR products with the expected band size were stored at 4°C for up to 48 h or frozen at -20°C for up to 7 days until sequencing.

### Nanopore Sequencing

Amplicons were purified using AMPure XP Reagent for PCR Purification (Beckman Coulter, Brea, CA, USA) using a 1:1 ratio of beads to amplicons and the remaining protocol carried out as per the manufacturer’s instructions. The rest of the procedure follows the Oxford Nanopore Technologies protocol “PCR barcoding (96) amplicons (SQK- LSK109)”. Library preparation was carried out using PCR barcodes (EXP-PBC096), ligation sequencing kit (SQK-LSK109), and flow cell priming kit (EXP-FLP002) per the manufacturer’s instructions. This enabled preparation of DNA libraries for sequencing on a MinION MK1C machine. Sequencing runs took between 8 and 48 h to generate a minimum of 20,000 reads per sample on R9.4.1 flow cells.

Once the MinION sequencing runs were stopped the fast5 files were processed using the high accuracy basecalling model (part of Guppy 4.3.4; (Oxford Nanopore Technologies, NA-a)) to generate fastq files for each barcode (minimum quality score of 7). The fastq files were then processed using a bioinformatics pipeline. Firstly, reads were trimmed using the program Cutadapt (version 3.2) to remove adapters, barcodes, and primer sequences (Martin, 2011). Trimmed reads were then aligned to reference genome sequences from Smith *et al*., (2020) or Kroneman *et al*., (2011) using Minimap2 (version 2.17) and Samtools (version 1.1) (Li, 2018; Danecek *et al*., 2021). Reads which aligned to a given reference with more than a 1000x coverage were error corrected using Canu (version 2.1.1) with a minimum overlap of 150bp, a minimum read length of 300bp and a minimum coverage of 30x (Koren *et al*., 2017). One error corrected read per reference was then saved into a file using Seqtk (version 1.3) and used as a consensus as previously described (Li, 2008). The process was repeated for each barcode, with consensus sequences from each barcode then aligned using MAFFT (version 7.475) (Katoh and Standley, 2013).

These alignments were then manually checked, and duplicate consensus sequences were removed. Sequences with a Hamming Dissimilarity distance (calculated within Unipro UGENE, Windows version 40 (Okonechnikov *et al*., 2012)) greater than 10 were classed as different sequences. Sequence reads were then aligned against the consensus sequences, and information such as coverage, alignment quality and the proportion of reads which aligned to a consensus were recorded, using Minimap2 and Samtools. The CPU version of Medaka (version 1.2.3) (Oxford Nanopore Technologies, NA-b) was then used to polish the consensus sequences. Consensus sequences were defined as being unique if they had a sequence dissimilarity of ≥5%, due to the possibility of errors from nanopore sequencing. Manual identification and removal of chimeric sequences present as a result of a sequencing artifact was then carried out. The G3 HEV and norovirus GII processing pipelines were very similar, differing only by the amplicon length, primer sequences and database of reference sequences. The code for these pipeline processes can be seen in Online Resources 2 and 3 (DOIs: 10.6084/m9.figshare.21900885; 10.6084/m9.figshare.21900900). The alignment files used can be seen in Online Resources 4 and 5 (DOIs: 10.6084/m9.figshare.21900903; 10.6084/m9.figshare.21900912). A minimum of 1,000 mapped reads were required to confirm the sequence was not present as an artifact due to barcode hopping or cross-contamination. All negative controls had less than 100 reads.

## Phylogenetic Analysis

For the norovirus GII phylogenetic analysis, excluding primer-derived sequences, sequence fragments of 302 bp were generated using the selected primers, however 20 bp of RNA-dependent RNA polymerase gene sequence was trimmed from the start of each sequence to enable comparison to capsid sequences from NCBI. This was done to prevent distortion of the phylogenetic analysis due to the presence of polymerase/capsid recombinants in the database. Reference sequences used for the phylogenetic analysis of the nucleotide sequencing data were retrieved from NCBI; 514 sequences for HEV genotypes 1-8, and 96 sequences for norovirus GII were downloaded and merged into two separate FASTA files, using BioEdit (Hall, 1999). The downloaded HEV sequences included sequences from humans, swine, deer, macaques and mongooses. The polished G3 HEV and norovirus GII consensus sequences obtained in the present study were then added to the appropriate FASTA files before alignment using Clustal Omega. The sequences were trimmed to be the same length as the sequenced amplicons (excluding primer-derived sequence and 20 bp of RNA-dependent RNA polymerase gene in the case of norovirus GII) and alignments were curated by eye. IQTREE was used to determine the most suitable evolutionary model for phylogenetic analysis, based on Bayesian Information Criteria scores (Nguyen *et al*., 2015). The substitution model selected was TIM2 with gamma distribution (TIM2 + G) for norovirus GII and G3 HEV. Phylogenetic trees were visualised in iTOL (Letunic and Bork, 2016).

HEV sequences were typed using the RIVM Hepatitis E Virus Genotyping Tool (https://www.rivm.nl/mpf/typingtool/hev/) and norovirus GII sequences were typed using the RIVM Norovirus Genotyping Tool (Kroneman *et al*., 2011). HEV sequences obtained from this study were uploaded to GenBank with accession numbers OQ918704 to OQ918713. Norovirus sequences were uploaded with accession numbers OQ913488 to OQ913500.

## Results

### Hepatitis E virus

Semi-nested PCR products with the expected size were obtained in 33 out of the 42 HEV positive samples by RT-qPCR selected for sequencing analysis. However, sequencing data were obtained from only 10 influent samples, with at least one HEV sequence per sample. No wastewater effluent samples yielded any HEV sequencing data. Online Resource 6 (DOI: 10.6084/m9.figshare.21900924) shows a box and whisker plot of the distribution of C_T_ values for samples which failed or succeeded to provide HEV sequences.

The number of mapped reads per HEV amplicon sequence ranged from 2,115 to 401,595 after sequences of incorrect length were removed. The number of reads and coverage data can be seen in Online Resource 7 (DOI: 10.6084/m9.figshare.22152383). Samples from which HEV sequencing data were obtained mostly yielded one consensus sequence irrespective of sequencing depth, but in a single sample two different HEV consensus sequences were obtained. The eleven HEV consensus sequences generated included ten different sequences; Wastewater seq1 and Wastewater seq6 (identified from different influent samples) were identical. All consensus sequences were genotyped as G3 using the RIVM Hepatitis E Virus Genotyping Tool, Wastewater seq1/seq6, Wastewater seq4 and Wastewater seq5 were subtyped as subtype G3c. The other sequences could not be subtyped due to weak phylogenetic support.

A nucleotide BLAST of the sequences showed the majority clustered most closely with GenBank HEV sequences detected in humans (Altschul *et al*., 1990). The results with the highest percentage identity can be seen in Online Resource 8 (DOI: 10.6084/m9.figshare.22300027).

**Fig. 1:**
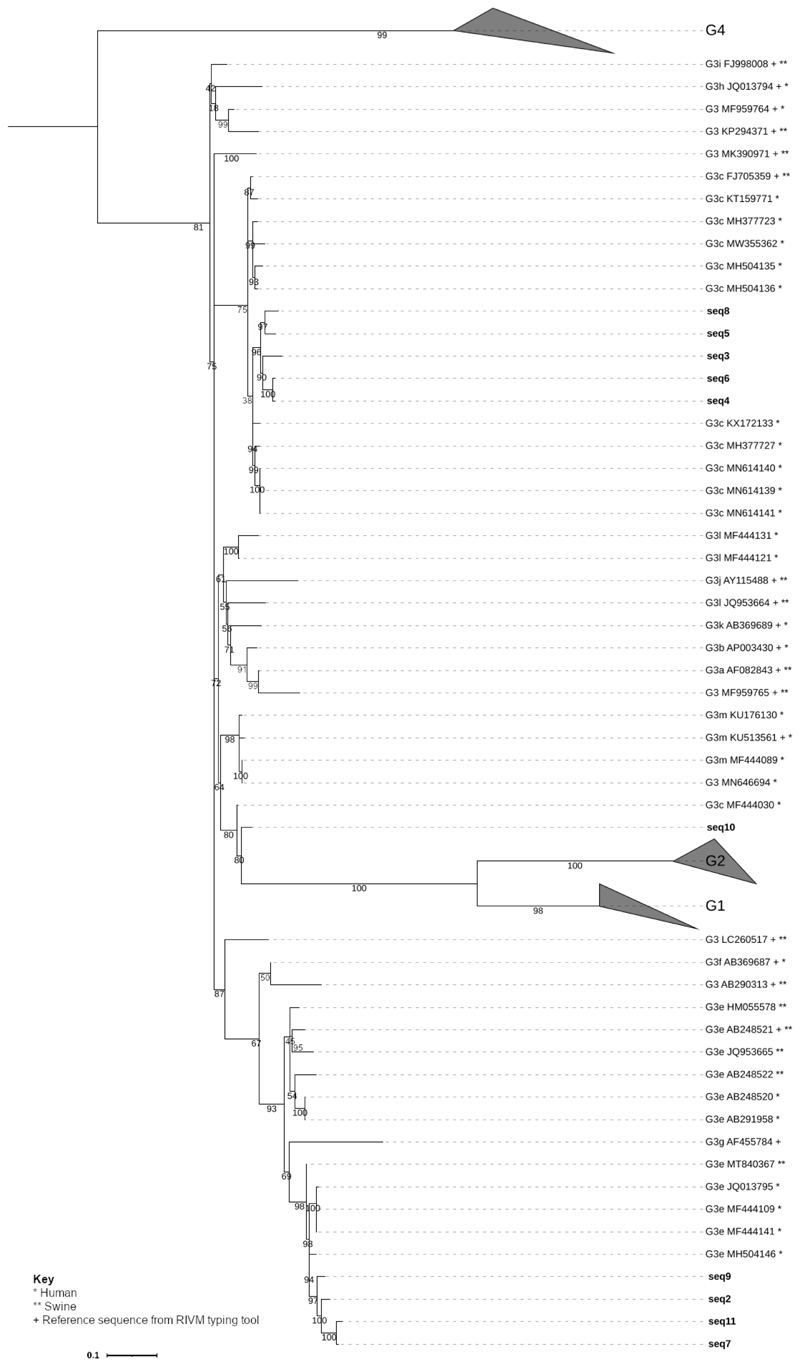
Pruned phylogenetic tree of HEV sequences. Pruned phylogenetic tree of a 215bp fragment from 514 HEV sequences, and ten unique sequences detected in the present study. Accession numbers can be seen in Online Resource 9 (DOI: 10.6084/m9.figshare.22584049). Pruning included sequences closely clustering with the consensus sequences and reference sequences from the RIVM genotyping tool (+). Genotypes other than G3 are collapsed. Bootstraps generated using ultrafast bootstrapping. The full tree, containing reference sequences from other human and animal hosts, can be seen in Online Resource 10 (DOI: 10.6084/m9.figshare.21900915). Wastewater_seq1 was not included in the tree as it was identical to Wastewater_seq6. The sequences from this study are shown with black bold labels. Published sequence labels contain * for human hosts or ** for swine hosts which the sequence was identified in. the host species it was identified in. The scale bar shows the length of branch that represents the substitutions per site of 0.1. Tree created using IQTREE and visualised in iTOL.

As only a few consensus sequences were successfully subtyped using the RIVM tool, phylogenetic analysis of the HEV sequences was performed to identify subtype clustering of the HEV consensus sequences. Fig. 1 shows the phylogenetic tree constructed using HEV consensus sequences from this study alongside previously published sequences. The tree showed that the consensus sequences obtained in this study cluster with HEV strains detected in humans and pigs. Note that the phylogenetic tree shown in Fig. 1 is pruned, the full tree includes HEV strains isolated from a wide variety of host species. These strains were all more distantly related to the consensus sequences than the human and pig-derived strains shown.

The phylogenetic tree shows that sequences 9, 2, 11 and 7 cluster with subtype G3e sequences, close to both HEV strains observed in humans and swine. Sequence 10 appears to cluster most closely with G3m, close to HEV strains detected in humans and swine. Sequences 3, 4, 5, 6 and 8 cluster together within G3c, most closely with HEV strains described in humans.

### Norovirus

Of the 42 influent and effluent wastewater samples which were selected for norovirus GII amplification by nPCR, 23 (12 influent and 11 effluent samples) yielded bands with the expected size and provided valid sequencing data (sequences with over 1,000 reads). Fifteen of the 42 samples did not amplify using the nPCR assay. One sample provided <1,000 reads and so was not analysed further. The remaining three samples did not provide norovirus sequences (despite showing an amplicon of the expected size by nPCR and inclusion in the library preparation). Online Resource 11 (DOI: 10.6084/m9.figshare.22299694) shows a box and whisker plot of the distribution of C_T_ values for samples which failed and succeeded.

The number of reads which mapped to norovirus GII reference sequences varied between 1,329 and 93,423 for the samples. The negative control produced no norovirus reads. The sequence mean depth per sample can be seen in Online Resource 12 (DOI: 10.6084/m9.figshare.21900921).

**Fig. 2:**
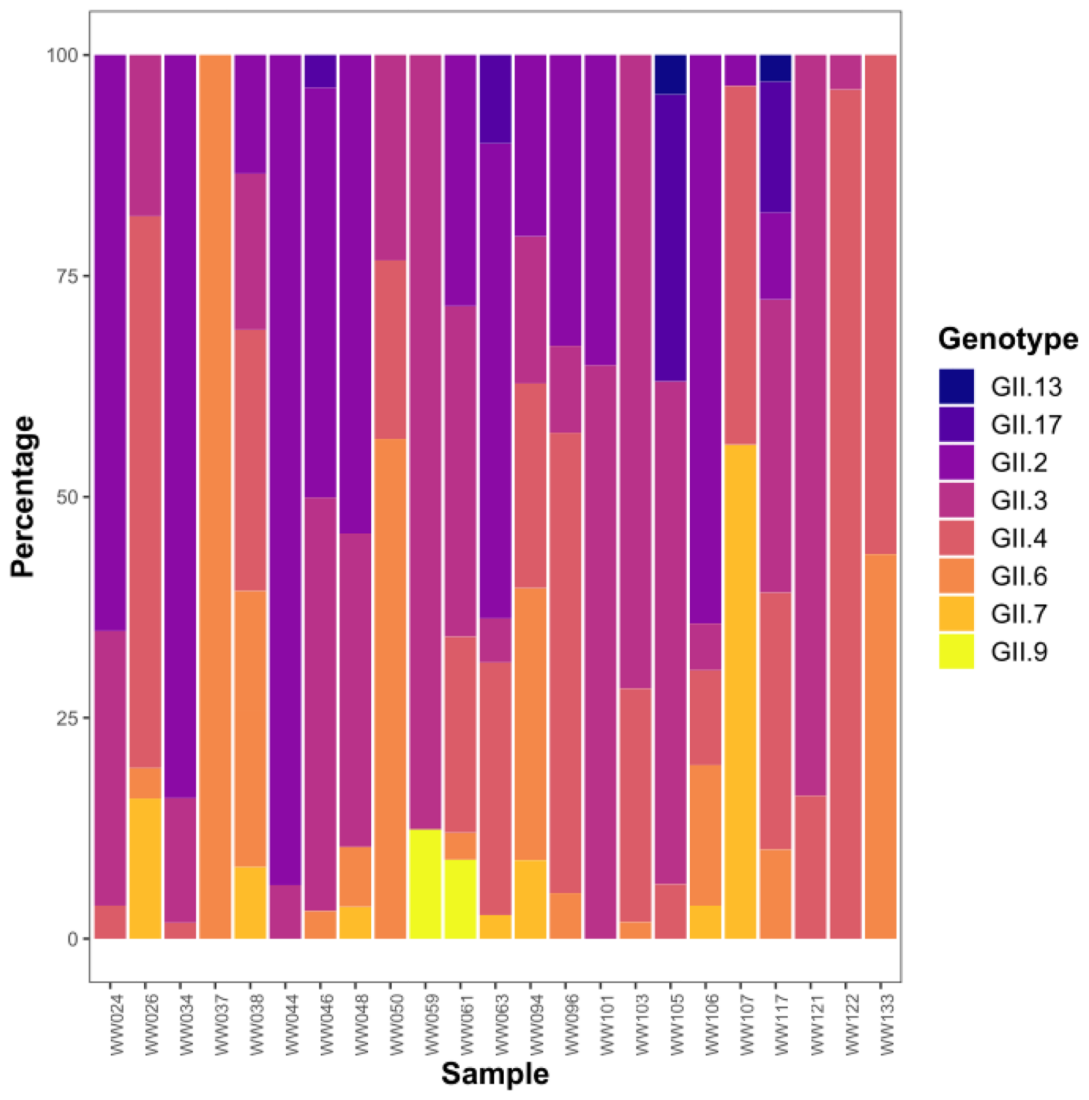
Percentage of reads attributed to each genotype of norovirus GII in wastewater samples. An alternative version suitable for the condition tritanopia can be seen in Online Resource 13 (DOI: 10.6084/m9.figshare.21900927). Figure created in R using package ggplot2.

Across the 23 samples that generated norovirus sequences, 93 consensus sequences in total were obtained from the wastewater samples, with 13 of these being unique sequences (where nucleotide sequence dissimilarity was greater than 5%). These 13 sequences represented eight genotypes as determined using the RIVM Norovirus Typing Tool: GII.2, GII.3, GII.4, GII.6, GII.7, GII.9, GII.13 and GII.17. The two different GII.4 consensus sequences were further identified by the Typing Tool as belonging to the Sydney 2012 strain. One of the wastewater samples contained only one genotype of norovirus GII whereas the remaining samples contained between two and six genotypes (Fig. 2).

Eleven of the unique sequences were found in multiple samples (up to a maximum of 20 samples). Between one and eight different norovirus GII consensus sequences were observed per sample. Multiple consensus sequences for the same genotypes were identified in eight samples (Table 4).

The genotype which was detected most frequently was GII.3, from 20 samples, followed by GII.4, in 17 different samples. Genotypes GII.2, GII.3, GII.4, GII.6, GII.7 and GII.17 were detected in the 12 influent samples. Genotypes GII.2, GII.3, GII.4, GII.6, GII.7, GII.9, GII.13 and GII.17 were detected in the effluent samples. The number of consensus sequences attributed to each genotype can be seen in Table 5. The most common genotype found in influent samples was GII.3 (in 12 samples), followed by GII.4 and GII.2 (9 samples each). The most common genotypes within effluent were GII.3 and GII.4 (both found in 8 samples). Sequences from GII.2 and GII.3 were detected in the influent and effluent of each WWTP which yielded sequencing results (five out of seven).

**Fig. 3:**
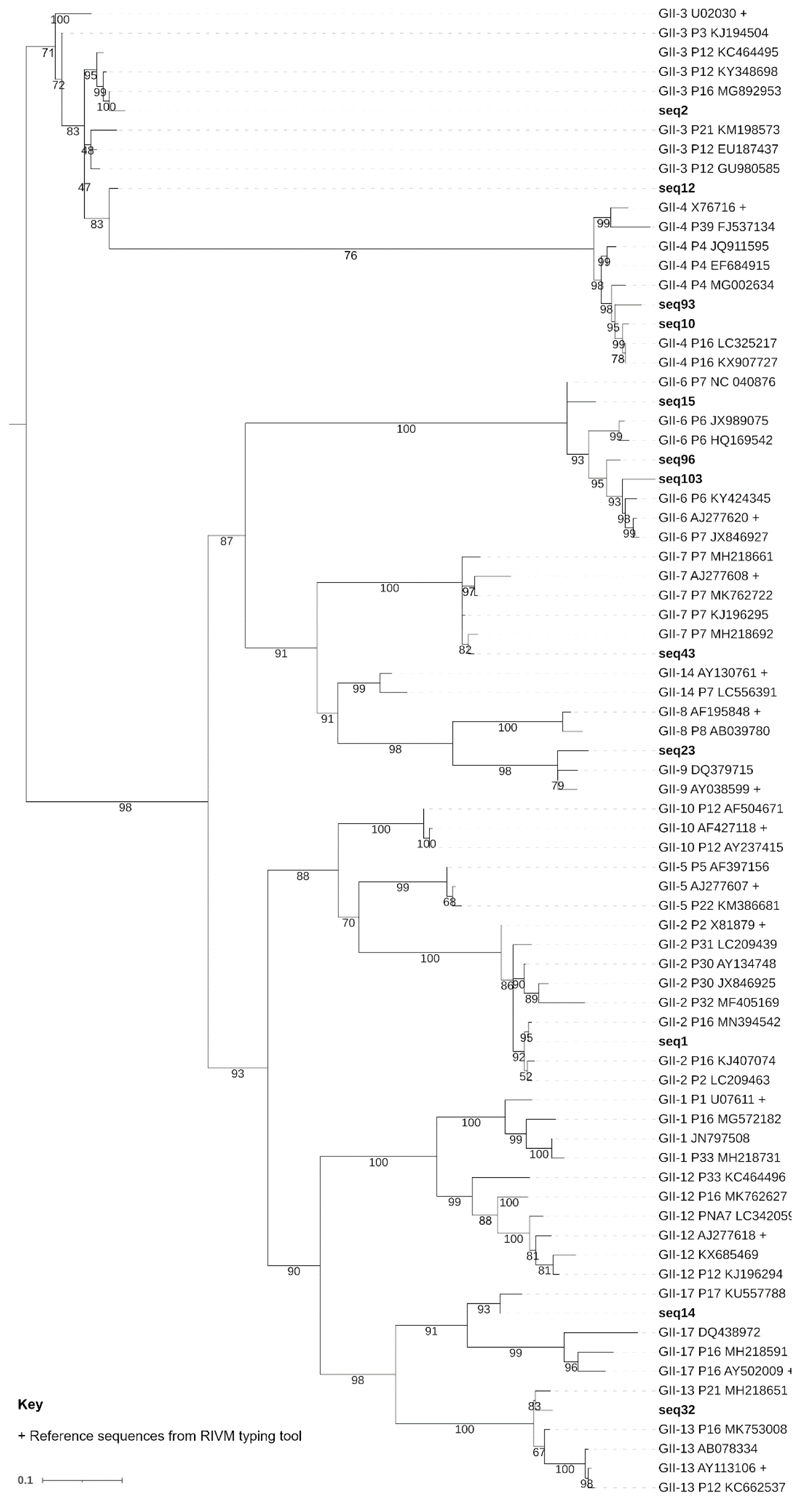
Pruned phylogenetic tree of norovirus GII sequences. Pruned phylogenetic tree constructed using a 282bp fragment of norovirus GII major capsid gene (VP1), showing reference sequences from the RIVM Norovirus genotyping tool (+). Amplicon sequences are labelled in bold text. Accession sequences available in Online Resource 14 (DOI: 10.6084/m9.figshare.22584079). Bootstrap support was >70% for all major genotype clades. The full phylogenetic tree, including reference sequences from other genotypes, can be viewed online in Online Resource 15 (DOI: 10.6084/m9.figshare.21900933). Only sequences obtained in the present study with a minimum nucleotide dissimilarity of 5% were included. The scale bar shows the length of branch that represents an amount of genetic change of 0.1. Figure created using IQTREE with visualisation in iTOL and editing in GIMP.

A Megablast search with default parameters was conducted to identify similar norovirus GII sequences in the GenBank nucleotide collection (nr/nt), revealing nucleotide sequence similarity values between 91 and 100%. Phylogenetic analysis of the VP1 sequences showed identical genotyping results to the ones obtained using the RIVM Norovirus Typing Tool (Fig. 3).

## Discussion

### HEV in wastewater

Of the 42 samples of wastewater influent and effluent analysed (all of which reported high C_T_ values during RT-qPCR for HEV), ten provided HEV sequencing data using the metabarcoding approach developed in the present study. The CT values of these samples ranged from 34 to 41, showing that even samples with low levels of virus can be sequenced using this technique, although several other samples with similar C_T_s could not be sequenced. It is possible that this was due to degradation of the HEV RNA within the wastewater samples.

The sequences from this study matched closely to G3c and G3e HEV sequences detected in humans in most cases but were also closely related to swine HEV sequences for others, suggesting that these species were the major sources of the viruses observed in the present study. This is unsurprising as the same HEV strains which circulate in swine also circulate in humans. However, it is possible that if there were more animal sequences publicly accessible that these results may have been less biased towards a link with HEV strains infecting humans or swine, as G3 HEV is capable of infecting many different animal hosts (Kenney, 2019). However, this can only be assessed when more HEV sequences from other animals become available. Considering that most of the WWTPs were fed primarily by human wastewater, it seems likely that most of the HEV sequences originated from human rather than animal sources. Some subtypes have become more dominant in the UK in the past two decades. Ijaz *et al*., (2013) and Grierson *et al*., (2015) showed the emergence of new phylotypes of G3 emerging within the UK, showing that HEV subtypes seemed to form two major clades, one of which had been dominant between 2003 and 2010 and one of which became more dominant from 2011 (Ijaz et al., 2013). Clade 1 includes subtypes 3e, 3f, 3g and clade 2 includes subtypes 3a, 3b, 3c, 3d, 3h, 3i, 3j (Ijaz *et al*., 2013; Smith *et al*., 2021).

In the UK in 2013, pigs were shown to generally be infected with viruses from clade 1, whilst humans were generally infected with viruses from clade 2 (Grierson et al., 2015); meanwhile HEV strains found in pigs from other European countries in the same time frame appeared to cluster with clade 2. A study on a limited number of UK infections in blood donors from 2018 – 2019 also showed most infections to belong to clade 2 (Smith et al., 2021). It appears that the virus subtypes identified within this study fall into both clades. As swine are thought to be the main reservoir of HEV, this may mean that swine HEV strains originally identified in both the UK and other European countries circulate within the UK. However, it is possible the swine data from the previous studies was too limited, and a phylogenetic comparison of the wastewater sequences to swine HEV strains in the UK was not possible (due to lack of UK HEV sequences). It was not possible to draw conclusions on whether these two major groups may be circulating more or less than previously reported due to small sample size, and there is no current data to suggest which subtypes were more dominant in the population in the UK in 2019.

The sequences obtained were from subtypes G3c and G3e, with one undetermined. Possible reasons for a lack of subtype diversity are that these were the subtypes circulating in the served populations of the WWTPs at the time, and that the WWTPs studied may not fully represent the population of the UK. There is no clinical data in the UK publicly available to compare the strains to for this time period, however, there was an outbreak of HEV in Italy at the end of 2019 where G3e and G3f were isolated from patients (Garbuglia *et al*., 2021), and strains isolated from patients in Spain in 2019 were from subtypes G3f and G3m (Muñoz-Chimeno *et al*., 2022). This means that G3e, G3f and G3m strains were actively circulating in Europe in 2019.

It is apparent that HEV presence in both wastewater and the community is likely to be infrequent in the UK, due to the low prevalence identified in previous studies, such as the identification of HEV in 3% of Scottish shellfish samples sold in a supermarket (O’Hara *et al*., 2018), and annual reports of clinically diagnosed HEV cases (Public Health England, 2019). However, despite low prevalence in these areas, HEV may contaminate the aquatic environment in the UK, most likely due to release of untreated human (and possibly animal) wastewater into water courses through combined sewer overflows (CSOs), which are allowed to spill into water courses during storm weather conditions. Considering that CSOs across the UK spilt into water courses for over 3 million hours in 2020, constituting 400,000 spills (Environment Agency, 2020; Laville, 2021), it is possible that HEV from human faecal sources regularly contaminates the aquatic environment. It seems less likely that HEV within treated effluent would be a large source of contamination for the aquatic environment as no effluent samples from this study provided any HEV sequence data, perhaps due to low copy number or RNA degradation. However, some effluent samples were RT-qPCR positive, so it cannot be ruled out.

### Norovirus GII in wastewater

Of the 42 wastewater samples with CT values between 27 and 39, 23 samples were successfully sequenced. The fifteen samples which did not amplify using the semi-nested PCR may have been too degraded to generate these amplicons. These samples contained eight different GII genotypes, with individual samples containing as many as six different genotypes. The norovirus GII sequences identified in this study were shown to have a nucleotide sequence similarity between 91 and 100% with sequences deposited in GenBank using nucleotide BLAST. Ten unique sequences were detected (all with >1,000 reads) in multiple individual samples, suggesting widely circulating strains, though this was not possible to confirm as the small amplicon length means that different strains may have produced identical sequence results. Surprisingly, GII.3 sequences were detected most commonly, within 20 samples; followed by GII.4 in 17 samples. GII.6 gave the highest number of unique sequences, with 4 identified across the samples. Two GII.4 unique sequences were also identified, and GII.4 has been the most frequently detected genotype in the world since the 1990s (Cannon *et al*., 2021), and has a high diversity (Parra *et al*., 2017). However, there were also two unique sequences from GII.3, as well as several other unique sequences from the other genotypes. This shows the high diversity of GII noroviruses present in wastewater, and therefore in the community, even in just a short five-month period. This agrees with findings reported by Ollivier *et al*., (2022), who also found a high level of norovirus diversity within oysters harvested between 2016 and 2018 across 12 different European countries. In the present study, GII.3 and GII.4 sequences were most common in effluent samples, but GII.3 was most common in influent samples (in 12 samples).

Sequence data was obtained from samples from five of seven WWTPs. GII.2 and GII.3 were the only genotypes which were detected at all five WWTPs. According to Public Health England (2020), these two genotypes were detected throughout 2019 in outbreaks that occurred in England or Wales.

Despite the abundance of different genotypes within the wastewater samples, clinical data from the UKHSA at the time of the study shows that though GII.4 and GII.6 made up a large proportion of cases, other detected genotypes such as GII.9 and GII.13 were not detected in cases from the public (Public Health England, 2020). This is likely to be explained by under-reporting of cases by the public, or by high frequency of asymptomatic illness, especially for certain genotypes, as norovirus cases are estimated to reach 3 million annually (Gherman *et al*., 2020), and the UK Health Security Agency reported only 6,172 symptomatic infections between 2018 and 2019 (Public Health England, 2021). It is possible that, with the finding of these potentially under-reported or asymptomatic genotypes of norovirus, that a similar situation may be occurring with norovirus GI and other genotypes of HEV, such as genotype 4. However, this would require further work to confirm as investigating norovirus GI and HEV G4 was outside of the scope of this study. These findings reinforce the application of sequencing environmental samples in the surveillance of human pathogenic viruses, as techniques such as these can enable detection of circulating strains which may not have been identified through clinical surveillance. Indeed, Fontenele *et al*., (2021) and many other groups utilised HTS technologies to sequence strains of SARS-CoV-2 from wastewater samples, providing an insight into circulating variants in the community.

A limitation of the study is that small fragment size and PCR-based methods may prevent possible detection of new HEV and norovirus GII variants (specifically if there were mutations in the primer regions or outside the amplicon region), and the apparent diversity observed was lower than the actual diversity. Another limitation was that the method was unable to detect norovirus recombinants as ORF1 and ORF2 were not sequenced simultaneously, and the nPCR assays used may have inherent biases towards certain genotypes or subtypes (Ollivier *et al*., 2022). However, sequencing of longer amplicons or whole genomes was unlikely to be successful due to the high C_T_ values for most samples, and it has been observed previously that longer amplicons than those of the RT-qPCR for detection can lead to lower sensitivity (Aprea *et al*., 2018; Grierson *et al*., 2015). However, the use of small amplicons has allowed sequencing data to be obtained to successfully genotype HEV and norovirus sequences. Another limitation is that Nanopore sequencing is known to have higher error rates than other sequencing platforms (such as Illumina), however Nanopore sequencing is still under active development, errors rates are reducing over time, and software is being constantly updated and refined to better deal with sequencing errors. Therefore, as Nanopore technologies improve, the results from these subtyping methods will also improve. Despite the limitations of this study, these methods provide a way to type HEV and norovirus from complex and difficult samples with high CT values where other methods such as shotgun sequencing or cloning/Sanger sequencing may not be feasible.

## Conclusion

In this study a metabarcoding approach using nanopore sequencing to genotype HEV and norovirus GII present in wastewater samples was successfully developed and applied. This could have several advantages for the sequencing of samples with low viral concentration in future studies (e.g. shellfish samples), and the use of the ONT platform for this method could enable greater portability and scalability of HEV and norovirus GII sequencing, as well as improving affordability over other sequencing platforms. The study has shown that HEV is present in wastewater in southern England and therefore that contamination of the aquatic environment with HEV could occur relatively frequently. It has also shown that many different genotypes of norovirus GII circulate simultaneously in wastewater, and that national surveillance of clinical norovirus cases may not be fully representative of all circulating genotypes within the population.

## Supplementary Material

Supplementary file 2

Supplementary file 6

Supplementary file 7

Supplementary file 8

Supplementary file 9

Supplementary file 10

Supplementary file 11

Supplementary file 12

Supplementary file 13

Supplementary file 14

Supplementary file 15

## Figures and Tables

**Table 1:** Characteristics of the WWTPs involved in the studies

WWTP number	Population equivalent	Dry weather flow (m^3^/day)	Treatment level (type)
**1**	33,822	9,450	Secondary (trickling filter)
**2**	166,837	40,486	Tertiary (UV)
**3**	178,531	55,000	Tertiary (UV)
**4**	166,931	47,700	Tertiary (sand filter and UV)
**5**	141,213	40,007	Secondary (activated sludge)
**6**	22,352	4,910	Tertiary (membrane filtration)
**7**	93,303	32,141	Secondary (aeration)

**Table 2:** Primer and probe sequences and details for the qRT-PCR methods

Target	Primer and Probe Sequences	Lower Limit of Detection	Reference
**HEV**	FWD: 5’-GGTGGTTTCTGGGGTGAC-3’ REV: 5’-AGGGGTTGGTTGGATGAA-3’ PROBE: 5’-TGATTCTCAGCCCTTCGC-3’ 5’-FAM 3’-MGB-NFQ	4 genome copies	Jothikumar et al., (2006); Garson et al., (2012)
**Norovirus GII**	FWD: 5’- ATGTTCAGRTGGATGAGRTTCTCWGA- 3’ REV: 5’-TCGACGCCATCTTCATTCACA-3’ PROBE: 5’-AGCACGTGGGAGGGCGATCG-3’ 5’-FAM 3’-TAMRA	1-10 genome copies (depending on strain)	Loisy et al., (2005); Kageyama et al., (2003)
**Mengo virus**	FWD: 5’-GCGGGTCCTGCCGAAAGT-3’ REV: 5’- GAAGTAACATATAGACAGACGCACAC- 3’ PROBE: 5’-ATCACATTACTGGCCGAAGC-3’ 5’-FAM 3’-MGB-NFQ	Unknown	Pintó, Costafreda and Bosch (2009)

**Table 3:** Nucleotide sequence of the primers used for amplification of HEV G3 and norovirus GII by semi-nested PCR.

Assay	Primer name and nucleotide sequences	Amplicon size (bp)	Target fragment size (bp)^b^	Reference
**Norovirus GII**	First round QNIF2D: 5’-ATGTTCAGRTGGATGAGRTTCTCWGA-3’ GIISKR: 5’-CCRCCNGCATRHCCRTTRTACAT-3’	378		Loisy *et al.,* (2005); Kojima *et al*., (2002)
	Second round GIISKF_T: 5’-**TTTCTGTTGGTGCTGATATTGC**CNTGGGAGGGCGATCGCAA -3’ GIISKR_T: 5’-**ACTTGCCTGTCGCTCTATCTTC**CCRCCNGCATRHCCRTTRTACAT-3’	344^a^	302	Kojima *et al*., (2002)
**HEV G3**	First round G3STF1: 5’-TGTTGCGCAGGTYTGTGT-3’ G3STR1: 5’-GCARCATAGGCARAARCACGA-3’	258		This study
	Second round G3STF2: 5’-**TTTCTGTTGGTGCTGATATTGC**TGTTGCGCAGGTYTGTGT-3’ G3STR2: 5’-**ACTTGCCTGTCGCTCTATCTTC**CATAGGCARAARCACGARGAA-3’	254^a^	215	This study

a The amplicon size given here does not include the primer adapters, which added 44 more base pairs. Sequences in bold are the primer adapters

b The amplicons are trimmed to remove the primer sequences to give the trimmed target fragment

**Table 4:** Number of unique sequences and genotypes observed for each sample.

Sample	Sample type	Number of unique sequences	Genotypes present (number of unique sequences)^a^
**SW024**	Influent	3	GII.2, GII.3, GII.4
**SW026**	Influent	4	GII.3, GII.4, GII.6, GII.7
**SW034**	Influent	3	GII.2, GII.3, GII.4
**SW037**	Effluent	1	GII.6
**SW038**	Influent	8	GII.2, GII.3, GII.4 (2), GII.6 (3), GII.7
**SW044**	Influent	2	GII.2, GII.3
**SW046**	Influent	5	GII.2, GII.3 (2), GII.6, GII.17
**SW048**	Influent	6	GII.2, GII.3, GII.6(3), GII.7
**SW050**	Influent	3	GII.3, GII.4, GII.6
**SW059**	Effluent	2	GII.3, GII.9
**SW061**	Effluent	5	GII.2, GII.3, GII.4, GII.6, GII.9
**SW063**	Effluent	5	GII.2, GII.3, GII.4, GII.7, GII.17
**SW094**	Influent	7	GII.2, GII.3, GII.4, GII.6 (3), GII.7
**SW096**	Influent	4	GII.2, GII.3, GII.4, GII.6
**SW101**	Effluent	2	GII.2, GII.3
**SW103**	Effluent	3	GII.3, GII.4, GII.6
**SW105**	Effluent	4	GII.3, GII.4, GII.13, GII.17
**SW106**	Influent	5	GII.2, GII.3, GII.4, GII.6, GII.7
**SW107**	Effluent	4	GII.2, GII.4 (2), GII.7
**SW117**	Effluent	6	GII.2, GII.3, GII.4, GII.6, GII.13, GII.7
**SW121**	Effluent	2	GII.3, GII.4
**SW122**	Influent	2	GII.3, GII.4
**SW133**	Effluent	2	GII.4, GII.6

a Each genotype was detected once in the sample unless otherwise specified in brackets.

**Table 5:** Sequences attributed to each norovirus GII genotype.

Genotype	Number of samples	Number of unique sequences
**GII.2**	14	1
**GII.3**	20	2
**GII.4**	17	2
**GII.6**	13	4
**GII.7**	7	1
**GII.9**	2	1
**GII.13**	2	1
**GII.17**	4	1
**TOTAL**	23	13

## Data Availability

See Online Resources for supplementary materials, including bioinformatics scripts. Sequences can be found in GenBank under accession numbers OQ913488 to OQ913500 and OQ918704 to OQ918713.

## Code availability

See Online Resources.
