# Supplementary material for: Metabarcoding of Hepatitis E virus genotype 3 and Norovirus GII from wastewater samples in England using nanopore sequencing
Source: Food Environ Virol. Author manuscript; Available in PMC 2023 Dec 1. (PMC7615314; doi:10.1007/s12560-023-09569-w)
Supplement: Supplementary file 2 [file EMS190417-supplement-Supplementary_file_2.html]

 
Norovirus Amplicon Data


Norovirus Amplicon Data

### Norovirus Amplicon Data

#### Primer sequences

- Short amplicons:

  - 1st round of PCR (378 bp):

    ```
    QNIF2D (forward) - ATGTTCAGRTGGATGAGRTTCTCWGA
    GIISKR (reverse)- CCRCCNGCATRHCCRTTRTACAT
    ```

    ```
    QNIF2D (forward) - ATGTTCAGRTGGATGAGRTTCTCWGA
    GIISKR (reverse)- CCRCCNGCATRHCCRTTRTACAT
    ```
  - 2nd round of PCR (343 bp):

    ```
    GIISKF (forward)  - CNTGGGAGGGCGATCGCAA
    GIISKR (reverse) - CCRCCNGCATRHCCRTTRTACAT
    ```

    ```
    GIISKF (forward)  - CNTGGGAGGGCGATCGCAA
    GIISKR (reverse) - CCRCCNGCATRHCCRTTRTACAT
    ```
- Long amplicons:

  - 1st round of PCR (1052 bp):

    ```
    NV4611 F (forward)  - CWGCAGCMCTDGAAATCATGG
    GIISKR (reverse) - CCRCCNGCATRHCCRTTRTACAT
    ```

    ```
    NV4611 F (forward)  - CWGCAGCMCTDGAAATCATGG
    GIISKR (reverse) - CCRCCNGCATRHCCRTTRTACAT
    ```
  - 2nd round of PCR (971 bp):

    ```
    NV4692F (forward)  - GTGTGRTKGATGTGGGTGACTT
    GIISKR (reverse) - CCRCCNGCATRHCCRTTRTACAT
    ```

    ```
    NV4692F (forward)  - GTGTGRTKGATGTGGGTGACTT
    GIISKR (reverse) - CCRCCNGCATRHCCRTTRTACAT
    ```

#### Download Norovirus Genome Sequences from NCBI

- Go to NCBI's taxonomic database:

  https://www.ncbi.nlm.nih.gov/taxonomy
- Search for a given species or taxon (e.g. 'norwalk virus'):
- Select the correct taxon from the search results and note the 'Taxonomic ID'

  https://www.ncbi.nlm.nih.gov/Taxonomy/Browser/wwwtax.cgi?mode=Info&id=11983&lvl=3&lin=f&keep=1&srchmode=1&unlock
- Search for sequences in NCBI's nucleotide database which are longer than 7,000bp, and which have been identified as a 'Norowalk Virus':

  ```
  txid11983[Organism:exp] AND 7000:100000[slen]
  ```

  ```
  txid11983[Organism:exp] AND 7000:100000[slen]
  ```
- Download the results:

  - Click on 'Send to' in the top right hand corner of the web browser.
  - Under 'Choose Destination' click on 'File'.
  - Under 'Format' select 'FASTA'.
- Go to the Downloads folder and rename the file 'Uncurated\_NCBI\_DB.fasta'.

#### Download a Curated Database of Norovirus Sequence Types

- Go to the following website:

  ```
  https://norovirus.ng.philab.cdc.gov/becerance.cgi
  ```

  ```
  https://norovirus.ng.philab.cdc.gov/becerance.cgi
  ```
- Copy the table and paste it into a MS Excel document.
- Reformat the table so that each row includes the accession number, the gene which was used for genotyping, the genotype and the name of the isolate.
- Download all the associated sequence data from NCBI and rename each sequence based on the original isolate which was used as a reference, the gene which was used for genotyping and the assigned genotype.
- Seperate out sequence data used for the VP1 and RdRP database.
- Seperately align sequence data which corresponds with the VP1 and RdRP database using MAFFT.
- View the alignments using the UGENE alignment viewer.
- Check coordinates in the alignment against a reference to establish what proportion of the VP1 and RdRP genes is represented.
- If necessary, trim regions which occur outside of the VP1 and RdRP genes.

#### Carry out high accuracy basecalling

- Find the folder which contains the sequencing data:

  ```
  $ cd ~/Documents/noro_11feb2021/no_sample/20210211_1624_MC-110340_0_FAO31769_1dc65ccf
  $ ls
  barcode_alignment_FAL41089_fce4b0dd.tsv
  drift_correction_FAO31769_fce4b0dd.csv
  duty_time_FAL41089_fce4b0dd.csv
  fast5_fail
  fast5_pass
  fastq_fail
  fastq_pass
  final_summary_FAO31769_fce4b0dd.txt
  mux_scan_data_FAO31769_fce4b0dd.csv
  report_FAL41089_20210211_1624_1dc65ccf.md
  report_FAL41089_20210211_1624_1dc65ccf.pdf
  sequencing_summary_FAO31769_fce4b0dd.txt
  throughput_FAL41089_fce4b0dd.csv
  ```

  ```
  $ cd ~/Documents/noro_11feb2021/no_sample/20210211_1624_MC-110340_0_FAO31769_1dc65ccf
  $ ls
  barcode_alignment_FAL41089_fce4b0dd.tsv
  drift_correction_FAO31769_fce4b0dd.csv
  duty_time_FAL41089_fce4b0dd.csv
  fast5_fail
  fast5_pass
  fastq_fail
  fastq_pass
  final_summary_FAO31769_fce4b0dd.txt
  mux_scan_data_FAO31769_fce4b0dd.csv
  report_FAL41089_20210211_1624_1dc65ccf.md
  report_FAL41089_20210211_1624_1dc65ccf.pdf
  sequencing_summary_FAO31769_fce4b0dd.txt
  throughput_FAL41089_fce4b0dd.csv
  ```
- Check the corresponding configuration file for a specific flowcell:

  ```
  $ guppy_basecaller --print_workflows
  ```

  ```
  $ guppy_basecaller --print_workflows
  ```
- Check your barcoding kit is supported by guppy:

  ```
  $ guppy_barcoder --print_kits
  ```

  ```
  $ guppy_barcoder --print_kits
  ```
- Use guppy to do high accuracy basecalling, adjusting the '-c' and '--barcode\_kits' parameters, as appropriate:

  ```
  $ guppy_basecaller \
  > -r -i ./ \
  > -s ./basecalled/ \
  > -c dna_r9.4.1_450bps_hac.cfg -x "cuda:0" \
  > --compress_fastq \
  > --num_callers 4 --gpu_runners_per_device 2 --chunks_per_runner 1500 --chunk_size 4000 \
  > --qscore_filtering \
  > --min_qscore 7 \
  > --trim_barcodes \
  > --num_barcoding_buffers 16 \
  > --barcode_kits SQK-LSK109
  ```

  ```
  $ guppy_basecaller \
  > -r -i ./ \
  > -s ./basecalled/ \
  > -c dna_r9.4.1_450bps_hac.cfg -x "cuda:0" \
  > --compress_fastq \
  > --num_callers 4 --gpu_runners_per_device 2 --chunks_per_runner 1500 --chunk_size 4000 \
  > --qscore_filtering \
  > --min_qscore 7 \
  > --trim_barcodes \
  > --num_barcoding_buffers 16 \
  > --barcode_kits SQK-LSK109
  ```

#### Check the output using NanoPlot

- Install NanoPlot:

  ```
  $ conda create --name=nanoplot nanoplot=1.32.1
  ```

  ```
  $ conda create --name=nanoplot nanoplot=1.32.1
  ```
- Use NanoPlot to calculate various summary statistics for reads shorter than 1200bp:

  ```
  $ cd basecalled
  $ NanoPlot -t 20 --summary sequencing_summary.txt --loglength -o summary-plots-log-transformed --barcoded --maxlength 1200
  ```

  ```
  $ cd basecalled
  $ NanoPlot -t 20 --summary sequencing_summary.txt --loglength -o summary-plots-log-transformed --barcoded --maxlength 1200
  ```
- Copy graphs which plot the read length against average quality scores to a separate folder and view the results:

  ```
  $ cd summary-plots-log-transformed
  $ mkdir LengthVsQualityScatterPlots
  $ cp *_LengthvsQualityScatterPlot_kde.png LengthVsQualityScatterPlots
  ```

  ```
  $ cd summary-plots-log-transformed
  $ mkdir LengthVsQualityScatterPlots
  $ cp *_LengthvsQualityScatterPlot_kde.png LengthVsQualityScatterPlots
  ```

#### Setup Computer for Analysing Data

- Find the folder which contains includes the fastq data output by the high accurary basecaller which had an average quality score of greater than seven:

  ```
  $ cd ~/Documents/noro_11feb2021/no_sample/20210211_1624_MC-110340_0_FAO31769_1dc65ccf/basecalled/pass/
  ```

  ```
  $ cd ~/Documents/noro_11feb2021/no_sample/20210211_1624_MC-110340_0_FAO31769_1dc65ccf/basecalled/pass/
  ```
- Create a new file to store a list of barcodes to analyse:

  ```
  $ nano shortAmpliconData.lst
  ```

  ```
  $ nano shortAmpliconData.lst
  ```

  - Include any barcodes for sequences amplified using the short amplicon primer set:

    ```
    ./barcode13
    ./barcode14
    ./barcode15
    ./barcode16
    ./barcode17
    ./barcode18
    ```

    ```
    ./barcode13
    ./barcode14
    ./barcode15
    ./barcode16
    ./barcode17
    ./barcode18
    ```
- Create a new file to store a list of barcodes to analyse:

  ```
  $ nano longAmpliconData.lst
  ```

  ```
  $ nano longAmpliconData.lst
  ```

  - Include any barcodes for sequences amplified using the long amplicon primer set:

    ```
    ./barcode21
    ./barcode22
    ./barcode23
    ./barcode24
    ```

    ```
    ./barcode21
    ./barcode22
    ./barcode23
    ./barcode24
    ```
- Combine fastq data from every barcode into a seperate file:

  ```
  folders=$(find ./ -maxdepth 1 -type d -name barcode\*)

  for folder in $folders
  do
          barcode=$(echo $folder | sed 's:./::g')
          echo $folder
          echo $barcode
          cat $folder/*fastq.gz > ${barcode}_merged.fastq.gz
  done
  ```

  ```
  folders=$(find ./ -maxdepth 1 -type d -name barcode\*)

  for folder in $folders
  do
          barcode=$(echo $folder | sed 's:./::g')
          echo $folder
          echo $barcode
          cat $folder/*fastq.gz > ${barcode}_merged.fastq.gz
  done
  ```

#### Trim Primer Sequences from Short Amplicons

- Install CutAdapt:

  ```
  $ conda create --name=cutadapt cutadapt=3.2
  ```

  ```
  $ conda create --name=cutadapt cutadapt=3.2
  ```
- Create a folder to store trimmed data:

  ```
  $ mkdir trim_seqs
  ```

  ```
  $ mkdir trim_seqs
  ```
- For every barcode amplified using the short primer set:

  - Trim reads which include the QNIF2D and GIISKR primers in the correct orientation, including the reverse complement.
  - Trim reads which include the GIISKF and GIISKR primers in the correct orientation, including the reverse complement.
  - If reads were not trimmed, output sequence data to a seperate file.
  - When identifying primers tolerate an error rate of 20 percent.
  - Save a summary of the results to a log file, for future reference.

    ```
    while read folderName;
    do
            barcode=$(echo $folderName | sed 's:./::g')
            echo $barcode

            eval "$(conda shell.bash hook)"
            conda activate cutadapt
            cutadapt -j20 \
                    -e 0.20 --revcomp \
                    -g ATGTTCAGRTGGATGAGRTTCTCWGA...ATGTAYAAYGGDYATGCNGGYGG \
                    -g CNTGGGAGGGCGATCGCAA...ATGTAYAAYGGDYATGCNGGYGG \
                    -o trim_seqs/${barcode}_trimmed.fastq.gz --untrimmed-output trim_seqs/${barcode}_untrimmed.fastq.gz ${barcode}_merged.fastq.gz > trim_seqs/${barcode}_trim.log 2>&1
    done<shortAmpliconData.lst
    ```

    ```
    while read folderName;
    do
            barcode=$(echo $folderName | sed 's:./::g')
            echo $barcode

            eval "$(conda shell.bash hook)"
            conda activate cutadapt
            cutadapt -j20 \
                    -e 0.20 --revcomp \
                    -g ATGTTCAGRTGGATGAGRTTCTCWGA...ATGTAYAAYGGDYATGCNGGYGG \
                    -g CNTGGGAGGGCGATCGCAA...ATGTAYAAYGGDYATGCNGGYGG \
                    -o trim_seqs/${barcode}_trimmed.fastq.gz --untrimmed-output trim_seqs/${barcode}_untrimmed.fastq.gz ${barcode}_merged.fastq.gz > trim_seqs/${barcode}_trim.log 2>&1
    done<shortAmpliconData.lst
    ```
- Check results from trimming barcodes amplified using the short primer set:

  ```
  $ head -n 20 trim_seqs/barcode13_trim.log
  This is cutadapt 3.2 with Python 3.8.6
  Command line parameters: -j20 -e 0.20 --revcomp -g ATGTTCAGRTGGATGAGRTTCTCWGA...ATGTAYAAYGGDYATGCNGGYGG -g CNTGGGAGGGCGATCGCAA...ATGTAYAAYGGDYATGCNGGYGG -o barcode13_trimmed.fastq.gz --untrimmed-output barcode13_untrimmed.fastq.gz barcode13_merged.fastq.gz
  Processing reads on 20 cores in single-end mode ...
  Finished in 3.26 s (11 µs/read; 5.28 M reads/minute).

  === Summary ===

  Total reads processed:                 286,755
  Reads with adapters:                   239,123 (83.4%)
  Reverse-complemented:                  115,178 (40.2%)
  Reads written (passing filters):       286,755 (100.0%)

  Total basepairs processed:   115,604,767 bp
  Total written (filtered):     89,473,360 bp (77.4%)

  === Adapter 3 ===

  Sequence: ATGTTCAGRTGGATGAGRTTCTCWGA...ATGTAYAAYGGDYATGCNGGYGG; Type: linked; Length: 26+23; 5' trimmed: 1747 times; 3' trimmed: 1747 times
  ; Reverse-complemented: 1206 times
  ```

  ```
  $ head -n 20 trim_seqs/barcode13_trim.log
  This is cutadapt 3.2 with Python 3.8.6
  Command line parameters: -j20 -e 0.20 --revcomp -g ATGTTCAGRTGGATGAGRTTCTCWGA...ATGTAYAAYGGDYATGCNGGYGG -g CNTGGGAGGGCGATCGCAA...ATGTAYAAYGGDYATGCNGGYGG -o barcode13_trimmed.fastq.gz --untrimmed-output barcode13_untrimmed.fastq.gz barcode13_merged.fastq.gz
  Processing reads on 20 cores in single-end mode ...
  Finished in 3.26 s (11 µs/read; 5.28 M reads/minute).

  === Summary ===

  Total reads processed:                 286,755
  Reads with adapters:                   239,123 (83.4%)
  Reverse-complemented:                  115,178 (40.2%)
  Reads written (passing filters):       286,755 (100.0%)

  Total basepairs processed:   115,604,767 bp
  Total written (filtered):     89,473,360 bp (77.4%)

  === Adapter 3 ===

  Sequence: ATGTTCAGRTGGATGAGRTTCTCWGA...ATGTAYAAYGGDYATGCNGGYGG; Type: linked; Length: 26+23; 5' trimmed: 1747 times; 3' trimmed: 1747 times
  ; Reverse-complemented: 1206 times
  ```
- For every barcode amplified using the long primer set:

  - Trim reads which include the NV4611F and GIISKR primers in the correct orientation, including any reverse complement sequences.
  - Trim reads which include the NV4692F and GIISKR primers in the correct orientation, including any reverse complement sequences.
  - If reads were not trimmed, output sequence data to a seperate file.
  - If the reads are shorter than 700bp, output to a seperate file.
  - When identifying primers tolerate an error rate of 20 percent.
  - Save a summary of the results to a log file, for future reference.

    ```
    while read folderName;
    do
            barcode=$(echo $folderName | sed 's:./::g')
            echo $barcode

            eval "$(conda shell.bash hook)"
            conda activate cutadapt
            cutadapt -j20 \
                    -e 0.20 --revcomp --minimum-length 700 \
                    -g CWGCAGCMCTDGAAATCATGG...ATGTAYAAYGGDYATGCNGGYGG \
                    -g GTGTGRTKGATGTGGGTGACTT...ATGTAYAAYGGDYATGCNGGYGG \
                    -o trim_seqs/${barcode}_trimmed.fastq.gz --too-short-output trim_seqs/${barcode}_tooShort.fastq.gz --untrimmed-output trim_seqs/${barcode}_untrimmed.fastq.gz ${barcode}_merged.fastq.gz > trim_seqs/${barcode}_trim.log 2>&1
    done<longAmpliconData.lst
    ```

    ```
    while read folderName;
    do
            barcode=$(echo $folderName | sed 's:./::g')
            echo $barcode

            eval "$(conda shell.bash hook)"
            conda activate cutadapt
            cutadapt -j20 \
                    -e 0.20 --revcomp --minimum-length 700 \
                    -g CWGCAGCMCTDGAAATCATGG...ATGTAYAAYGGDYATGCNGGYGG \
                    -g GTGTGRTKGATGTGGGTGACTT...ATGTAYAAYGGDYATGCNGGYGG \
                    -o trim_seqs/${barcode}_trimmed.fastq.gz --too-short-output trim_seqs/${barcode}_tooShort.fastq.gz --untrimmed-output trim_seqs/${barcode}_untrimmed.fastq.gz ${barcode}_merged.fastq.gz > trim_seqs/${barcode}_trim.log 2>&1
    done<longAmpliconData.lst
    ```
- Check results from trimming barcodes amplified using the long primer set:

  ```
  $ head -n 20 trim_seqs/barcode21_trim.log
  This is cutadapt 3.2 with Python 3.8.6
  Command line parameters: -j20 -e 0.20 --revcomp --minimum-length 700 -g CWGCAGCMCTDGAAATCATGG...ATGTAYAAYGGDYATGCNGGYGG -g GTGTGRTKGATGTGGGTGACTT...ATGTAYAAYGGDYATGCNGGYGG -o barcode21_trimmed.fastq.gz --too-short-output barcode21_tooShort.fastq.gz --untrimmed-output barcode21_untrimmed.fastq.gz barcode21_merged.fastq.gz
  Processing reads on 20 cores in single-end mode ...
  Finished in 8.48 s (16 µs/read; 3.74 M reads/minute).

  === Summary ===

  Total reads processed:                 527,985
  Reads with adapters:                   470,290 (89.1%)
  Reverse-complemented:                  234,126 (44.3%)
  Reads that were too short:             313,951 (59.5%)
  Reads written (passing filters):       527,985 (100.0%)

  Total basepairs processed:   302,299,297 bp
  Total written (filtered):    277,207,480 bp (91.7%)

  === Adapter 3 ===

  Sequence: CWGCAGCMCTDGAAATCATGG...ATGTAYAAYGGDYATGCNGGYGG; Type: linked; Length: 21+23; 5' trimmed: 120079 times; 3' trimmed: 120079 times
  ; Reverse-complemented: 68877 times
  ```

  ```
  $ head -n 20 trim_seqs/barcode21_trim.log
  This is cutadapt 3.2 with Python 3.8.6
  Command line parameters: -j20 -e 0.20 --revcomp --minimum-length 700 -g CWGCAGCMCTDGAAATCATGG...ATGTAYAAYGGDYATGCNGGYGG -g GTGTGRTKGATGTGGGTGACTT...ATGTAYAAYGGDYATGCNGGYGG -o barcode21_trimmed.fastq.gz --too-short-output barcode21_tooShort.fastq.gz --untrimmed-output barcode21_untrimmed.fastq.gz barcode21_merged.fastq.gz
  Processing reads on 20 cores in single-end mode ...
  Finished in 8.48 s (16 µs/read; 3.74 M reads/minute).

  === Summary ===

  Total reads processed:                 527,985
  Reads with adapters:                   470,290 (89.1%)
  Reverse-complemented:                  234,126 (44.3%)
  Reads that were too short:             313,951 (59.5%)
  Reads written (passing filters):       527,985 (100.0%)

  Total basepairs processed:   302,299,297 bp
  Total written (filtered):    277,207,480 bp (91.7%)

  === Adapter 3 ===

  Sequence: CWGCAGCMCTDGAAATCATGG...ATGTAYAAYGGDYATGCNGGYGG; Type: linked; Length: 21+23; 5' trimmed: 120079 times; 3' trimmed: 120079 times
  ; Reverse-complemented: 68877 times
  ```

#### Align Trimmed Reads Against an Uncurated Database

- Install Minimap2 and Samtools:

  ```
  $ conda create --name=minimap2 minimap2=2.17 samtools=1.11
  ```

  ```
  $ conda create --name=minimap2 minimap2=2.17 samtools=1.11
  ```
- Create a folder to store alignments:

  ```
  $ mkdir Minimap_Vs_All_Ref_Seqs/
  ```

  ```
  $ mkdir Minimap_Vs_All_Ref_Seqs/
  ```
- For every barcode:

  - Align sequence data against full genome sequences from NCBI.
  - Summarise information on coverage, the average quality of the alignment and the proportion of sequences which aligned against a reference.

    ```
    folders=$(find ./ -maxdepth 1 -type d -name barcode\*)

    for folder in $folders
    do
            barcode=$(echo $folder | sed 's:./::g')
            echo $folder
            echo $barcode

            eval "$(conda shell.bash hook)"
            conda activate minimap2

            minimap2 --secondary=no -t18 -ax map-ont /mnt/Storage1/db/Norovirus_Uncurated_NCBI_DB.fasta trim_seqs/${barcode}_trimmed.fastq.gz |\
                    samtools sort -@18 -o Minimap_Vs_All_Ref_Seqs/alignment_${barcode}.bam

            samtools index -@ 18 Minimap_Vs_All_Ref_Seqs/alignment_${barcode}.bam
            samtools flagstat Minimap_Vs_All_Ref_Seqs/alignment_${barcode}.bam > Minimap_Vs_All_Ref_Seqs/alignment_${barcode}_flagstat.report
            samtools coverage Minimap_Vs_All_Ref_Seqs/alignment_${barcode}.bam | awk '$4 >0' | sort -rnk4 > Minimap_Vs_All_Ref_Seqs/alignment_${barcode}_coverage.report

    done
    ```

    ```
    folders=$(find ./ -maxdepth 1 -type d -name barcode\*)

    for folder in $folders
    do
            barcode=$(echo $folder | sed 's:./::g')
            echo $folder
            echo $barcode

            eval "$(conda shell.bash hook)"
            conda activate minimap2

            minimap2 --secondary=no -t18 -ax map-ont /mnt/Storage1/db/Norovirus_Uncurated_NCBI_DB.fasta trim_seqs/${barcode}_trimmed.fastq.gz |\
                    samtools sort -@18 -o Minimap_Vs_All_Ref_Seqs/alignment_${barcode}.bam

            samtools index -@ 18 Minimap_Vs_All_Ref_Seqs/alignment_${barcode}.bam
            samtools flagstat Minimap_Vs_All_Ref_Seqs/alignment_${barcode}.bam > Minimap_Vs_All_Ref_Seqs/alignment_${barcode}_flagstat.report
            samtools coverage Minimap_Vs_All_Ref_Seqs/alignment_${barcode}.bam | awk '$4 >0' | sort -rnk4 > Minimap_Vs_All_Ref_Seqs/alignment_${barcode}_coverage.report

    done
    ```
- Around 80 - 90 percent of the sequencing data should align against one of the norovirus genomes published on NCBI.
- If not, think carefully about the experimental details, and check the length of amplicons using NanoPlot.
- A similar proportion of reads should align against the curated database in the next step of the analysis.
- If not, your curated database may be incomplete.

#### Align Trimmed Reads Against a Curated Database of genotypes associated with the VP1 gene

- Create a folder to store alignments:

  ```
  $ mkdir Minimap_Vs_VP1_Curated_Seqs
  ```

  ```
  $ mkdir Minimap_Vs_VP1_Curated_Seqs
  ```
- For every barcode:

  - Align sequence data against a curate database of genotypes associated with the VP1 gene.
  - Summarise information on coverage, the average quality of the alignment and the proportion of sequences which aligned against a reference.
  - Note, this is the same as the last script, with a different reference database and output folder.

    ```
    folders=$(find ./ -maxdepth 1 -type d -name barcode\*)

    for folder in $folders
    do
            barcode=$(echo $folder | sed 's:./::g')
            echo $folder
            echo $barcode

            eval "$(conda shell.bash hook)"
            conda activate minimap2

            minimap2 --secondary=no -t18 -ax map-ont /mnt/Storage1/db/VP1_Sequences_V4_Subalignment.fasta trim_seqs/${barcode}_trimmed.fastq.gz |\
                    samtools sort -@18 -o Minimap_Vs_VP1_Curated_Seqs/alignment_${barcode}.bam

            samtools index -@ 18 Minimap_Vs_VP1_Curated_Seqs/alignment_${barcode}.bam
            samtools flagstat Minimap_Vs_VP1_Curated_Seqs/alignment_${barcode}.bam > Minimap_Vs_VP1_Curated_Seqs/alignment_${barcode}_flagstat.report
            samtools coverage Minimap_Vs_VP1_Curated_Seqs/alignment_${barcode}.bam | awk '$4 >0' | sort -rnk4 > Minimap_Vs_VP1_Curated_Seqs/alignment_${barcode}_coverage.report

    done
    ```

    ```
    folders=$(find ./ -maxdepth 1 -type d -name barcode\*)

    for folder in $folders
    do
            barcode=$(echo $folder | sed 's:./::g')
            echo $folder
            echo $barcode

            eval "$(conda shell.bash hook)"
            conda activate minimap2

            minimap2 --secondary=no -t18 -ax map-ont /mnt/Storage1/db/VP1_Sequences_V4_Subalignment.fasta trim_seqs/${barcode}_trimmed.fastq.gz |\
                    samtools sort -@18 -o Minimap_Vs_VP1_Curated_Seqs/alignment_${barcode}.bam

            samtools index -@ 18 Minimap_Vs_VP1_Curated_Seqs/alignment_${barcode}.bam
            samtools flagstat Minimap_Vs_VP1_Curated_Seqs/alignment_${barcode}.bam > Minimap_Vs_VP1_Curated_Seqs/alignment_${barcode}_flagstat.report
            samtools coverage Minimap_Vs_VP1_Curated_Seqs/alignment_${barcode}.bam | awk '$4 >0' | sort -rnk4 > Minimap_Vs_VP1_Curated_Seqs/alignment_${barcode}_coverage.report

    done
    ```
- I also wrote a script using R which reads files with the coverage.report suffix and plots the results. There are a few parts of the script which are specifically tailered to this particular database, but if you are familar with R, it might help.

#### Align Trimmed Reads Against a Curated Database of genotypes associated with the RdRP gene

- Create a folder to store alignments:

  ```
  $ mkdir Minimap_Vs_RdRP_Curated_Seqs
  ```

  ```
  $ mkdir Minimap_Vs_RdRP_Curated_Seqs
  ```
- For every barcode:

  - Align sequence data against a curate database of genotypes associated with the RdRP gene.
  - Summarise information on coverage, the average quality of the alignment and the proportion of sequences which aligned against a reference.
  - Note, this is the same as the last two scripts, with a different reference database and output folder.

    ```
    folders=$(find ./ -maxdepth 1 -type d -name barcode\*)

    for folder in $folders
    do
            barcode=$(echo $folder | sed 's:./::g')
            echo $folder
            echo $barcode

            eval "$(conda shell.bash hook)"
            conda activate minimap2

            minimap2 --secondary=no -t18 -ax map-ont /mnt/Storage1/db/RdRP_Sequences_V4_Subalignment.fasta trim_seqs/${barcode}_trimmed.fastq.gz |\
                    samtools sort -@18 -o Minimap_Vs_RdRP_Curated_Seqs/alignment_${barcode}.bam

            samtools index -@ 18 Minimap_Vs_RdRP_Curated_Seqs/alignment_${barcode}.bam
            samtools flagstat Minimap_Vs_RdRP_Curated_Seqs/alignment_${barcode}.bam > Minimap_Vs_RdRP_Curated_Seqs/alignment_${barcode}_flagstat.report
            samtools coverage Minimap_Vs_RdRP_Curated_Seqs/alignment_${barcode}.bam | awk '$4 >0' | sort -rnk4 > Minimap_Vs_RdRP_Curated_Seqs/alignment_${barcode}_coverage.report

    done
    ```

    ```
    folders=$(find ./ -maxdepth 1 -type d -name barcode\*)

    for folder in $folders
    do
            barcode=$(echo $folder | sed 's:./::g')
            echo $folder
            echo $barcode

            eval "$(conda shell.bash hook)"
            conda activate minimap2

            minimap2 --secondary=no -t18 -ax map-ont /mnt/Storage1/db/RdRP_Sequences_V4_Subalignment.fasta trim_seqs/${barcode}_trimmed.fastq.gz |\
                    samtools sort -@18 -o Minimap_Vs_RdRP_Curated_Seqs/alignment_${barcode}.bam

            samtools index -@ 18 Minimap_Vs_RdRP_Curated_Seqs/alignment_${barcode}.bam
            samtools flagstat Minimap_Vs_RdRP_Curated_Seqs/alignment_${barcode}.bam > Minimap_Vs_RdRP_Curated_Seqs/alignment_${barcode}_flagstat.report
            samtools coverage Minimap_Vs_RdRP_Curated_Seqs/alignment_${barcode}.bam | awk '$4 >0' | sort -rnk4 > Minimap_Vs_RdRP_Curated_Seqs/alignment_${barcode}_coverage.report

    done
    ```
- I also wrote a script using R which reads files with the coverage.report suffix and plots the results. There are a few parts of the script which are specifically tailered to this particular database, but if you are familar with R, it might help.

#### Creating a Set of Consensus Sequences for each Genotype

- Create folders to store the initial results after grouping reads into specific genotypes and then assembling each group of reads:

  ```
  $ mkdir assemble_long_amplicons
  $ mkdir assemble_long_amplicons/intermediate_files/
  $ mkdir assemble_short_amplicons
  $ mkdir assemble_short_amplicons/intermediate_files/
  ```

  ```
  $ mkdir assemble_long_amplicons
  $ mkdir assemble_long_amplicons/intermediate_files/
  $ mkdir assemble_short_amplicons
  $ mkdir assemble_short_amplicons/intermediate_files/
  ```
- Install canu:

  ```
  $ conda create --name=canu canu=2.1.1
  ```

  ```
  $ conda create --name=canu canu=2.1.1
  ```
- For every barcode amplified using the short primer set:

  - Compile a list of reference sequences which have sufficient coverage.
  - Sufficient coverage in this case is based on the number of reads which align against a specific reference.
  - References are included if more than 1000 reads align with a specific barcode.
  - For each reference with sufficient coverage:
    - Extract any reads which aligned against the reference, and save them in a seperate fastq file.
    - Use canu to carry out error correction of the reads.
    - Specify the expected genome size as 1kbp (using the 'genomeSize=1k' parameter)
    - Make sure any read is error corrected, regardless of length (using the 'corOutCoverage=1000000' parameter)
    - Don't use the job submission system on POD (using the 'useGrid=false' parameter)
    - Use a minimum read length of 300bp (using the 'minReadLength=300' parameter)
    - The minimum overlap between any two reads in a pairwise alignment should be 150bp (using the 'minOverlapLength=150' parameter)
    - When error correcting the reads a minimum coverage of 30x should be used (using the 'corMinCoverage=30' parameter)
    - Use parameters recommened for flowcells incorporating the R9.4 sequencing chemistry (the line beginning with 'corMhapOptions').

      ```
      while read folderName;
      do
              barcode=$(echo $folderName | sed 's:./::g')
              echo $barcode
              references=$(awk '$4>1000' Minimap_Vs_VP1_Curated_Seqs/alignment_${barcode}_coverage.report | cut -f1 | grep -v "\#")

              for reference in $references
              do
                      eval "$(conda shell.bash hook)"
                      conda activate minimap2
                      samtools view Minimap_Vs_VP1_Curated_Seqs/alignment_${barcode}.bam $reference | awk '{ print "@" $1 "\n" $10 "\n+\n" $11 }' > assemble_short_amplicons/intermediate_files/${barcode}_${reference}.fastq

                      eval "$(conda shell.bash hook)"
                      conda activate canu
                      canu -p ${barcode}_${reference}_assembly -d assemble_short_amplicons/intermediate_files/${barcode}_${reference}_assembly -correct \
                      -nanopore assemble_short_amplicons/intermediate_files/${barcode}_${reference}.fastq \
                      genomeSize=1k \
                      corOutCoverage=1000000 \
                      useGrid=false \
                      maxThreads=20 maxMemory=100 \
                      minReadLength=300 minOverlapLength=150 corMinCoverage=30 \
                      corMhapOptions="--threshold 0.8 --ordered-sketch-size 1000 --ordered-kmer-size 14" correctedErrorRate=0.105 > assemble_short_amplicons/intermediate_files/${barcode}_${reference}_assembly.log 2>&1


              done
      done<shortAmpliconData.lst
      ```

      ```
      while read folderName;
      do
              barcode=$(echo $folderName | sed 's:./::g')
              echo $barcode
              references=$(awk '$4>1000' Minimap_Vs_VP1_Curated_Seqs/alignment_${barcode}_coverage.report | cut -f1 | grep -v "\#")

              for reference in $references
              do
                      eval "$(conda shell.bash hook)"
                      conda activate minimap2
                      samtools view Minimap_Vs_VP1_Curated_Seqs/alignment_${barcode}.bam $reference | awk '{ print "@" $1 "\n" $10 "\n+\n" $11 }' > assemble_short_amplicons/intermediate_files/${barcode}_${reference}.fastq

                      eval "$(conda shell.bash hook)"
                      conda activate canu
                      canu -p ${barcode}_${reference}_assembly -d assemble_short_amplicons/intermediate_files/${barcode}_${reference}_assembly -correct \
                      -nanopore assemble_short_amplicons/intermediate_files/${barcode}_${reference}.fastq \
                      genomeSize=1k \
                      corOutCoverage=1000000 \
                      useGrid=false \
                      maxThreads=20 maxMemory=100 \
                      minReadLength=300 minOverlapLength=150 corMinCoverage=30 \
                      corMhapOptions="--threshold 0.8 --ordered-sketch-size 1000 --ordered-kmer-size 14" correctedErrorRate=0.105 > assemble_short_amplicons/intermediate_files/${barcode}_${reference}_assembly.log 2>&1


              done
      done<shortAmpliconData.lst
      ```
- For every barcode amplified using the long primer set:

  - Do the same as above, except using a minimum read length of 900bp and a minimum overlap of 400bp (using the 'minReadLength=900 minOverlapLength=400' parameters)

    ```
    while read folderName;
    do
            barcode=$(echo $folderName | sed 's:./::g')
            echo $barcode
            references=$(awk '$4>1000' Minimap_Vs_VP1_Curated_Seqs/alignment_${barcode}_coverage.report | cut -f1 | grep -v "\#")

            for reference in $references
            do
                    eval "$(conda shell.bash hook)"
                    conda activate minimap2
                    samtools view Minimap_Vs_VP1_Curated_Seqs/alignment_${barcode}.bam $reference | awk '{ print "@" $1 "\n" $10 "\n+\n" $11 }' > assemble_long_amplicons/intermediate_files/${barcode}_${reference}.fastq

                    eval "$(conda shell.bash hook)"
                    conda activate canu
                    canu -p ${barcode}_${reference}_assembly -d assemble_long_amplicons/intermediate_files/${barcode}_${reference}_assembly -correct \
                    -nanopore assemble_long_amplicons/intermediate_files/${barcode}_${reference}.fastq \
                    genomeSize=1k \
                    corOutCoverage=1000000 \
                    useGrid=false \
                    maxThreads=20 maxMemory=100 \
                    minReadLength=900 minOverlapLength=400 corMinCoverage=30 \
                    corMhapOptions="--threshold 0.8 --ordered-sketch-size 1000 --ordered-kmer-size 14" correctedErrorRate=0.105 > assemble_long_amplicons/intermediate_files/${barcode}_${reference}_assembly.log 2>&1


            done
    done<shortAmpliconData.lst
    ```

    ```
    while read folderName;
    do
            barcode=$(echo $folderName | sed 's:./::g')
            echo $barcode
            references=$(awk '$4>1000' Minimap_Vs_VP1_Curated_Seqs/alignment_${barcode}_coverage.report | cut -f1 | grep -v "\#")

            for reference in $references
            do
                    eval "$(conda shell.bash hook)"
                    conda activate minimap2
                    samtools view Minimap_Vs_VP1_Curated_Seqs/alignment_${barcode}.bam $reference | awk '{ print "@" $1 "\n" $10 "\n+\n" $11 }' > assemble_long_amplicons/intermediate_files/${barcode}_${reference}.fastq

                    eval "$(conda shell.bash hook)"
                    conda activate canu
                    canu -p ${barcode}_${reference}_assembly -d assemble_long_amplicons/intermediate_files/${barcode}_${reference}_assembly -correct \
                    -nanopore assemble_long_amplicons/intermediate_files/${barcode}_${reference}.fastq \
                    genomeSize=1k \
                    corOutCoverage=1000000 \
                    useGrid=false \
                    maxThreads=20 maxMemory=100 \
                    minReadLength=900 minOverlapLength=400 corMinCoverage=30 \
                    corMhapOptions="--threshold 0.8 --ordered-sketch-size 1000 --ordered-kmer-size 14" correctedErrorRate=0.105 > assemble_long_amplicons/intermediate_files/${barcode}_${reference}_assembly.log 2>&1


            done
    done<shortAmpliconData.lst
    ```

#### Pick Consensus Sequences from Error Corrected Reads Output by Canu

- Create a folder to store the consensus sequences:

  ```
  $ mkdir assemble_short_amplicons/contigs/
  $ mkdir assemble_long_amplicons/contigs/
  ```

  ```
  $ mkdir assemble_short_amplicons/contigs/
  $ mkdir assemble_long_amplicons/contigs/
  ```
- Install seqtk and mafft:

  ```
  $ conda create --name=seqtk seqtk=1.3
  $ conda create --name=mafft mafft=7.475
  ```

  ```
  $ conda create --name=seqtk seqtk=1.3
  $ conda create --name=mafft mafft=7.475
  ```
- For every barcode amplified using the short primer set:

  - For each reference associated with a newly assembled consensus sequence:
    - Randomly pick the first error corrected sequence longer than 288bp.
    - Rename the consensus sequence based on the barcode and name of the reference which was used as a template.
    - Save all the consensus sequences for a given barcode into a single file.
    - Align all the consensus sequences from a given barcode using MAFFT.

      ```
      cd assemble_short_amplicons

      while read folderName;
          do
          barcode=$(echo $folderName | sed 's:./::g')
          echo $barcode
          references=$(awk '$4>1000' ../Minimap_Vs_VP1_Curated_Seqs/alignment_${barcode}_coverage.report | cut -f1 | grep -v "\#")

          rm contigs/${barcode}_seqs.fasta
          for reference in $references
          do
                  eval "$(conda shell.bash hook)"
                  conda activate seqtk
                  seqtk seq -L 288 intermediate_files/${barcode}_${reference}_assembly/${barcode}_${reference}_assembly.correctedReads.fasta.gz | head -n2 > contigs/${barcode}_${reference}_contigs.fasta
                  contigName=$(head -n1 contigs/${barcode}_${reference}_contigs.fasta | sed 's/>//g')
                  sed -i "s/${contigName}/${barcode}_${reference}/g" contigs/${barcode}_${reference}_contigs.fasta
                  cat contigs/${barcode}_${reference}_contigs.fasta >> contigs/${barcode}_seqs.fasta
          done

          eval "$(conda shell.bash hook)"
          conda activate mafft

          mafft --adjustdirection --reorder contigs/${barcode}_seqs.fasta > contigs/${barcode}_aligned.fasta
      done<../shortAmpliconData.lst
      ```

      ```
      cd assemble_short_amplicons

      while read folderName;
          do
          barcode=$(echo $folderName | sed 's:./::g')
          echo $barcode
          references=$(awk '$4>1000' ../Minimap_Vs_VP1_Curated_Seqs/alignment_${barcode}_coverage.report | cut -f1 | grep -v "\#")

          rm contigs/${barcode}_seqs.fasta
          for reference in $references
          do
                  eval "$(conda shell.bash hook)"
                  conda activate seqtk
                  seqtk seq -L 288 intermediate_files/${barcode}_${reference}_assembly/${barcode}_${reference}_assembly.correctedReads.fasta.gz | head -n2 > contigs/${barcode}_${reference}_contigs.fasta
                  contigName=$(head -n1 contigs/${barcode}_${reference}_contigs.fasta | sed 's/>//g')
                  sed -i "s/${contigName}/${barcode}_${reference}/g" contigs/${barcode}_${reference}_contigs.fasta
                  cat contigs/${barcode}_${reference}_contigs.fasta >> contigs/${barcode}_seqs.fasta
          done

          eval "$(conda shell.bash hook)"
          conda activate mafft

          mafft --adjustdirection --reorder contigs/${barcode}_seqs.fasta > contigs/${barcode}_aligned.fasta
      done<../shortAmpliconData.lst
      ```
- For every barcode amplified using the long primer set:

  - For each reference associated with a newly assembled consensus sequence:
    - Randomly pick the first error corrected sequence longer than 910bp.
    - Rename the consensus sequence based on the barcode and name of the reference which was used as a template.
    - Save all the consensus sequences for a given barcode into a single file.
    - Align all the consensus sequences from a given barcode using MAFFT.

      ```
      cd assemble_long_amplicons

      while read folderName;
      do
              barcode=$(echo $folderName | sed 's:./::g')
              echo $barcode
              references=$(awk '$4>1000' ../Minimap_Vs_VP1_Curated_Seqs/alignment_${barcode}_coverage.report | cut -f1 | grep -v "\#")

              rm contigs/${barcode}_seqs.fasta
              for reference in $references
              do
                      eval "$(conda shell.bash hook)"
                      conda activate seqtk
                      seqtk seq -L 910 intermediate_files/${barcode}_${reference}_assembly/${barcode}_${reference}_assembly.correctedReads.fasta.gz | head -n2 > contigs/${barcode}_${reference}_contigs.fasta
                      contigName=$(head -n1 contigs/${barcode}_${reference}_contigs.fasta | sed 's/>//g')
                      sed -i "s/${contigName}/${barcode}_${reference}/g" contigs/${barcode}_${reference}_contigs.fasta
                      cat contigs/${barcode}_${reference}_contigs.fasta >> contigs/${barcode}_seqs.fasta
              done

              eval "$(conda shell.bash hook)"
              conda activate mafft

              mafft --adjustdirection --reorder contigs/${barcode}_seqs.fasta > contigs/${barcode}_aligned.fasta
      done<../longAmpliconData.lst
      ```

      ```
      cd assemble_long_amplicons

      while read folderName;
      do
              barcode=$(echo $folderName | sed 's:./::g')
              echo $barcode
              references=$(awk '$4>1000' ../Minimap_Vs_VP1_Curated_Seqs/alignment_${barcode}_coverage.report | cut -f1 | grep -v "\#")

              rm contigs/${barcode}_seqs.fasta
              for reference in $references
              do
                      eval "$(conda shell.bash hook)"
                      conda activate seqtk
                      seqtk seq -L 910 intermediate_files/${barcode}_${reference}_assembly/${barcode}_${reference}_assembly.correctedReads.fasta.gz | head -n2 > contigs/${barcode}_${reference}_contigs.fasta
                      contigName=$(head -n1 contigs/${barcode}_${reference}_contigs.fasta | sed 's/>//g')
                      sed -i "s/${contigName}/${barcode}_${reference}/g" contigs/${barcode}_${reference}_contigs.fasta
                      cat contigs/${barcode}_${reference}_contigs.fasta >> contigs/${barcode}_seqs.fasta
              done

              eval "$(conda shell.bash hook)"
              conda activate mafft

              mafft --adjustdirection --reorder contigs/${barcode}_seqs.fasta > contigs/${barcode}_aligned.fasta
      done<../longAmpliconData.lst
      ```

#### Check Alignment of Consensus Sequences for each Barcode

- Download files with the 'aligned.fasta' suffix from the 'contigs' folder to your laptop.
- Open the alignment for each set of consensus sequences using the UGENE software on your laptop.
- Where necessary, when opening the alignment, select the 'Join sequences into alignment and open in multiple alignment viewer' option.
- Under the context menu go to:

  `Actions -> Statistics -> Generate Distance Matrix`
- Select the 'Hamming dissimilarity' distance algorithm.
- Make sure the profile mode is set to 'Count' and 'Exclude gaps' is ticked.
- Click on the 'Generate' button.
- Use the distance matrix to identify duplicates, defined for our purposes as sequences from the same sample or barcode which are seperated by a distance of less than 10.
- Make sure the final set of consensus sequences are unique, and there are no duplicates.
- If you decide to delete any duplicates, make sure they are removed from files with the 'seqs.fasta' suffix in the contigs folder:

  ```
  $ cd assemble_long_amplicons
  $ nano contigs/barcode21_seqs.fasta
  ```

  ```
  $ cd assemble_long_amplicons
  $ nano contigs/barcode21_seqs.fasta
  ```
- In addition, whilst editing files with the 'seqs.fasta' suffix assign each consensus sequence a unique number (i.e. change the name of the sequence to start with seq1, seq2, seq3, etc)

#### Align Sequence Data against Consensus Sequences

- Create a folder to store the new set of alignments:

  ```
  $ mkdir assemble_short_amplicons/align_against_consensus_seqs
  $ mkdir assemble_long_amplicons/align_against_consensus_seqs
  ```

  ```
  $ mkdir assemble_short_amplicons/align_against_consensus_seqs
  $ mkdir assemble_long_amplicons/align_against_consensus_seqs
  ```
- For every barcode amplified using the short primer set:

  - Align sequence data against the consensus sequences.
  - Summarise information on coverage, the average quality of the alignment and the proportion of sequences which aligned against a reference.

  ```
  cd assemble_short_amplicons

  while read folder;
  do
      barcode=$(echo $folder | sed 's:./::g')
      echo $folder
      echo $barcode
      eval "$(conda shell.bash hook)"
      conda activate minimap2

      minimap2 --secondary=no -t18 -ax map-ont ./contigs/${barcode}_seqs.fasta ../${barcode}_merged.fastq.gz |\
      samtools sort -@18 -o align_against_consensus_seqs/alignment_${barcode}.bam

      samtools index -@ 18 align_against_consensus_seqs/alignment_${barcode}.bam
      samtools flagstat align_against_consensus_seqs/alignment_${barcode}.bam > align_against_consensus_seqs/alignment_${barcode}_flagstat.report
      samtools coverage align_against_consensus_seqs/alignment_${barcode}.bam | awk '$4 >0' | sort -rnk4 > align_against_consensus_seqs/alignment_${barcode}_coverage.report

  done<../shortAmpliconData.lst
  ```

  ```
  cd assemble_short_amplicons

  while read folder;
  do
      barcode=$(echo $folder | sed 's:./::g')
      echo $folder
      echo $barcode
      eval "$(conda shell.bash hook)"
      conda activate minimap2

      minimap2 --secondary=no -t18 -ax map-ont ./contigs/${barcode}_seqs.fasta ../${barcode}_merged.fastq.gz |\
      samtools sort -@18 -o align_against_consensus_seqs/alignment_${barcode}.bam

      samtools index -@ 18 align_against_consensus_seqs/alignment_${barcode}.bam
      samtools flagstat align_against_consensus_seqs/alignment_${barcode}.bam > align_against_consensus_seqs/alignment_${barcode}_flagstat.report
      samtools coverage align_against_consensus_seqs/alignment_${barcode}.bam | awk '$4 >0' | sort -rnk4 > align_against_consensus_seqs/alignment_${barcode}_coverage.report

  done<../shortAmpliconData.lst
  ```
- For every barcode amplified using the long primer set:

  - Align sequence data against the consensus sequences.
  - Summarise information on coverage, the average quality of the alignment and the proportion of sequences which aligned against a reference.
  - This is exactly the same as before, with different samples.

  ```
  cd assemble_long_amplicons

  while read folder;
  do
          barcode=$(echo $folder | sed 's:./::g')
          echo $folder
          echo $barcode
          eval "$(conda shell.bash hook)"
          conda activate minimap2
          minimap2 --secondary=no -t18 -ax map-ont ./contigs/${barcode}_seqs.fasta ../${barcode}_trimmed.fastq.gz |\
                  samtools sort -@18 -o align_against_consensus_seqs/alignment_${barcode}.bam

          samtools index -@ 18 align_against_consensus_seqs/alignment_${barcode}.bam
          samtools flagstat align_against_consensus_seqs/alignment_${barcode}.bam > align_against_consensus_seqs/alignment_${barcode}_flagstat.report
          samtools coverage align_against_consensus_seqs/alignment_${barcode}.bam | awk '$4 >0' | sort -rnk4 > align_against_consensus_seqs/alignment_${barcode}_coverage.report

  done<../longAmpliconData.lst
  ```

  ```
  cd assemble_long_amplicons

  while read folder;
  do
          barcode=$(echo $folder | sed 's:./::g')
          echo $folder
          echo $barcode
          eval "$(conda shell.bash hook)"
          conda activate minimap2
          minimap2 --secondary=no -t18 -ax map-ont ./contigs/${barcode}_seqs.fasta ../${barcode}_trimmed.fastq.gz |\
                  samtools sort -@18 -o align_against_consensus_seqs/alignment_${barcode}.bam

          samtools index -@ 18 align_against_consensus_seqs/alignment_${barcode}.bam
          samtools flagstat align_against_consensus_seqs/alignment_${barcode}.bam > align_against_consensus_seqs/alignment_${barcode}_flagstat.report
          samtools coverage align_against_consensus_seqs/alignment_${barcode}.bam | awk '$4 >0' | sort -rnk4 > align_against_consensus_seqs/alignment_${barcode}_coverage.report

  done<../longAmpliconData.lst
  ```
- Check alignment results and compare results to those that were initially produced via alignment against the reference database.

#### Use Medaka to Polish the Consensus Sequences

- Create a folder to store results from medaka:

  ```
  $ mkdir assemble_short_amplicons/medaka_consensus/
  $ mkdir assemble_short_amplicons/medaka_contigs/
  $ mkdir assemble_long_amplicons/medaka_consensus/
  $ mkdir assemble_long_amplicons/medaka_contigs/
  ```

  ```
  $ mkdir assemble_short_amplicons/medaka_consensus/
  $ mkdir assemble_short_amplicons/medaka_contigs/
  $ mkdir assemble_long_amplicons/medaka_consensus/
  $ mkdir assemble_long_amplicons/medaka_contigs/
  ```
- Install the CPU version of medaka:

  ```
  conda create --name=medaka_cpu medaka=1.2.3 tensorflow=2.2.0=mkl_py36h5a57954_0
  ```

  ```
  conda create --name=medaka_cpu medaka=1.2.3 tensorflow=2.2.0=mkl_py36h5a57954_0
  ```
- For every barcode amplified using the short primer set:

  - Run medaka\_consensus, using the sequencing data and consensus sequences as an input.

  ```
  cd assemble_short_amplicons

  while read folder;
  do
          barcode=$(echo $folder | sed 's:./::g')
          echo $folder
          echo $barcode
          eval "$(conda shell.bash hook)"
          conda activate medaka_cpu
          medaka_consensus -i ../${barcode}_trimmed.fastq.gz -d contigs/${barcode}_seqs.fasta -o medaka_consensus/${barcode}_consensus -t 20 -m r941_min_high_g360
          cp medaka_consensus/${barcode}_consensus/consensus.fasta medaka_contigs/${barcode}_polished_seqs.fasta

  done<../shortAmpliconData.lst
  ```

  ```
  cd assemble_short_amplicons

  while read folder;
  do
          barcode=$(echo $folder | sed 's:./::g')
          echo $folder
          echo $barcode
          eval "$(conda shell.bash hook)"
          conda activate medaka_cpu
          medaka_consensus -i ../${barcode}_trimmed.fastq.gz -d contigs/${barcode}_seqs.fasta -o medaka_consensus/${barcode}_consensus -t 20 -m r941_min_high_g360
          cp medaka_consensus/${barcode}_consensus/consensus.fasta medaka_contigs/${barcode}_polished_seqs.fasta

  done<../shortAmpliconData.lst
  ```
- For every barcode amplified using the long primer set:

  - Run medaka consensus, using the sequencing data and consensus sequences as an input (exactly the same as before, with different samples)

  ```
  cd assemble_long_amplicons

  while read folder;
  do
          barcode=$(echo $folder | sed 's:./::g')
          echo $folder
          echo $barcode
          eval "$(conda shell.bash hook)"
          conda activate medaka_cpu
          medaka_consensus -i ../${barcode}_trimmed.fastq.gz -d contigs/${barcode}_seqs.fasta -o medaka_consensus/${barcode}_consensus -t 20 -m r941_min_high_g360
          cp medaka_consensus/${barcode}_consensus/consensus.fasta medaka_contigs/${barcode}_polished_seqs.fasta

  done<../longAmpliconData.lst
  ```

  ```
  cd assemble_long_amplicons

  while read folder;
  do
          barcode=$(echo $folder | sed 's:./::g')
          echo $folder
          echo $barcode
          eval "$(conda shell.bash hook)"
          conda activate medaka_cpu
          medaka_consensus -i ../${barcode}_trimmed.fastq.gz -d contigs/${barcode}_seqs.fasta -o medaka_consensus/${barcode}_consensus -t 20 -m r941_min_high_g360
          cp medaka_consensus/${barcode}_consensus/consensus.fasta medaka_contigs/${barcode}_polished_seqs.fasta

  done<../longAmpliconData.lst
  ```

#### Use Medaka to Call Variants

- Create a folder to store results from medaka:

  ```
  $ mkdir assemble_short_amplicons/medaka_variant_calling/
  $ mkdir assemble_long_amplicons/medaka_variant_calling/
  ```

  ```
  $ mkdir assemble_short_amplicons/medaka_variant_calling/
  $ mkdir assemble_long_amplicons/medaka_variant_calling/
  ```
- For each set of consensus sequences amplified using the short primer set:

  - Align sequencing data against the polished consensus sequences.
  - Summarise information on coverage, the average quality of the alignment and the proportion of sequences which aligned against a reference.
  - Use the medaka\_variant tool to identify any variants present in the alignment.

    ```
    cd assemble_short_amplicons

    while read folder;
    do
            barcode=$(echo $folder | sed 's:./::g')
            echo $folder
            echo $barcode

            eval "$(conda shell.bash hook)"
            conda activate minimap2
            minimap2 --secondary=no -t18 -ax map-ont ./medaka_contigs/${barcode}_polished_seqs.fasta ../${barcode}_trimmed.fastq.gz |\
                    samtools sort -@18 -o medaka_variant_calling/alignment_${barcode}.bam

            samtools index -@ 18 medaka_variant_calling/alignment_${barcode}.bam
            samtools flagstat medaka_variant_calling/alignment_${barcode}.bam > medaka_variant_calling/alignment_${barcode}_flagstat.report
            samtools coverage medaka_variant_calling/alignment_${barcode}.bam | awk '$4 >0' | sort -rnk4 > medaka_variant_calling/alignment_${barcode}_coverage.report

            eval "$(conda shell.bash hook)"
            conda activate medaka_cpu
            medaka_variant -i medaka_variant_calling/alignment_${barcode}.bam -m r941_min_high_g360 -s r941_min_high_g360 -f medaka_contigs/${barcode}_polished_seqs.fasta -o medaka_variant_calling/${barcode}_variants
    done<../shortAmpliconData.lst
    ```

    ```
    cd assemble_short_amplicons

    while read folder;
    do
            barcode=$(echo $folder | sed 's:./::g')
            echo $folder
            echo $barcode

            eval "$(conda shell.bash hook)"
            conda activate minimap2
            minimap2 --secondary=no -t18 -ax map-ont ./medaka_contigs/${barcode}_polished_seqs.fasta ../${barcode}_trimmed.fastq.gz |\
                    samtools sort -@18 -o medaka_variant_calling/alignment_${barcode}.bam

            samtools index -@ 18 medaka_variant_calling/alignment_${barcode}.bam
            samtools flagstat medaka_variant_calling/alignment_${barcode}.bam > medaka_variant_calling/alignment_${barcode}_flagstat.report
            samtools coverage medaka_variant_calling/alignment_${barcode}.bam | awk '$4 >0' | sort -rnk4 > medaka_variant_calling/alignment_${barcode}_coverage.report

            eval "$(conda shell.bash hook)"
            conda activate medaka_cpu
            medaka_variant -i medaka_variant_calling/alignment_${barcode}.bam -m r941_min_high_g360 -s r941_min_high_g360 -f medaka_contigs/${barcode}_polished_seqs.fasta -o medaka_variant_calling/${barcode}_variants
    done<../shortAmpliconData.lst
    ```
- For each set of consensus sequences amplified using the long primer set:

  - Align sequencing data against the polished consensus sequences.
  - Summarise information on coverage, the average quality of the alignment and the proportion of sequences which aligned against a reference.
  - Use the medaka\_variant tool to identify any variants present in the alignment.
  - This is exactly the same as before, with different samples.

    ```
    cd assemble_long_amplicons

    while read folder;
    do
            barcode=$(echo $folder | sed 's:./::g')
            echo $folder
            echo $barcode

            eval "$(conda shell.bash hook)"
            conda activate minimap2
            minimap2 --secondary=no -t18 -ax map-ont ./medaka_contigs/${barcode}_polished_seqs.fasta ../${barcode}_trimmed.fastq.gz |\
                    samtools sort -@18 -o medaka_variant_calling/alignment_${barcode}.bam

            samtools index -@ 18 medaka_variant_calling/alignment_${barcode}.bam
            samtools flagstat medaka_variant_calling/alignment_${barcode}.bam > medaka_variant_calling/alignment_${barcode}_flagstat.report
            samtools coverage medaka_variant_calling/alignment_${barcode}.bam | awk '$4 >0' | sort -rnk4 > medaka_variant_calling/alignment_${barcode}_coverage.report

            eval "$(conda shell.bash hook)"
            conda activate medaka_cpu
            medaka_variant -i medaka_variant_calling/alignment_${barcode}.bam -m r941_min_high_g360 -s r941_min_high_g360 -f medaka_contigs/${barcode}_polished_seqs.fasta -o medaka_variant_calling/${barcode}_variants
    done<../longAmpliconData.lst
    ```

    ```
    cd assemble_long_amplicons

    while read folder;
    do
            barcode=$(echo $folder | sed 's:./::g')
            echo $folder
            echo $barcode

            eval "$(conda shell.bash hook)"
            conda activate minimap2
            minimap2 --secondary=no -t18 -ax map-ont ./medaka_contigs/${barcode}_polished_seqs.fasta ../${barcode}_trimmed.fastq.gz |\
                    samtools sort -@18 -o medaka_variant_calling/alignment_${barcode}.bam

            samtools index -@ 18 medaka_variant_calling/alignment_${barcode}.bam
            samtools flagstat medaka_variant_calling/alignment_${barcode}.bam > medaka_variant_calling/alignment_${barcode}_flagstat.report
            samtools coverage medaka_variant_calling/alignment_${barcode}.bam | awk '$4 >0' | sort -rnk4 > medaka_variant_calling/alignment_${barcode}_coverage.report

            eval "$(conda shell.bash hook)"
            conda activate medaka_cpu
            medaka_variant -i medaka_variant_calling/alignment_${barcode}.bam -m r941_min_high_g360 -s r941_min_high_g360 -f medaka_contigs/${barcode}_polished_seqs.fasta -o medaka_variant_calling/${barcode}_variants
    done<../longAmpliconData.lst
    ```
