# Supplementary material for: Metabarcoding of Hepatitis E virus genotype 3 and Norovirus GII from wastewater samples in England using nanopore sequencing
Source: Food Environ Virol. Author manuscript; Available in PMC 2023 Dec 1. (PMC7615314; doi:10.1007/s12560-023-09569-w)
Supplement: Supplementary file 6 [file EMS190417-supplement-Supplementary_file_6.docx]

Metabarcoding of Hepatitis E virus genotype 3 and Norovirus GII from wastewater samples in England using nanopore sequencing

Samantha Treagus^1,2^, James Lowther^1^, Ben Longdon^2^, William Gaze^3^, Craig Baker-Austin^1^, David Ryder^1^, Frederico M. Batista^1^

*Author for editorial correspondence:*

Samantha Treagus

UK Health Security Agency

Manor Farm Road

Porton Down

Wiltshire

SP4 0JG

United Kingdom

Email: samantha.treagus2@ukhsa.gov.uk

ORCID iD: 0000-0002-1905-9024

**
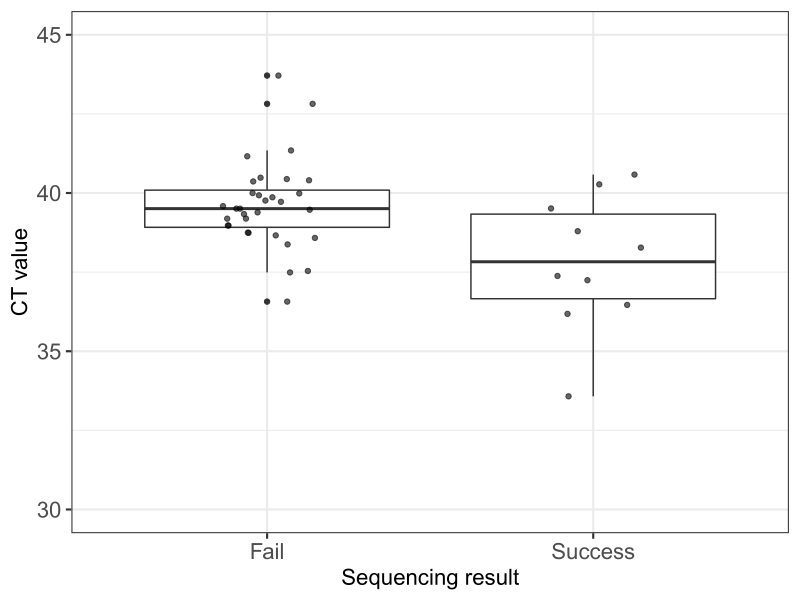
Online Resource 6**

**Fig. 1** A box and whisker plot of the distribution of HEV C_T_ values

The distribution of C_T_ values from qRT-PCR which either succeeded or failed to yield sequencing data. Figure created in R.
