# Supplementary material for: Metabarcoding of Hepatitis E virus genotype 3 and Norovirus GII from wastewater samples in England using nanopore sequencing
Source: Food Environ Virol. Author manuscript; Available in PMC 2023 Dec 1. (PMC7615314; doi:10.1007/s12560-023-09569-w)
Supplement: Supplementary file 7 [file EMS190417-supplement-Supplementary_file_7.docx]

Metabarcoding of Hepatitis E virus genotype 3 and Norovirus GII from wastewater samples in England using nanopore sequencing

Samantha Treagus^1,2^, James Lowther^1^, Ben Longdon^2^, William Gaze^3^, Craig Baker-Austin^1^, David Ryder^1^, Frederico M. Batista^1^

*Author for editorial correspondence:*

Samantha Treagus

UK Health Security Agency

Manor Farm Road

Porton Down

Wiltshire

SP4 0JG

United Kingdom

Email: samantha.treagus2@ukhsa.gov.uk

ORCID iD: 0000-0002-1905-9024

**Online Resource 7**

**Table 1** Mapped reads and level of coverage for each HEV amplicon sequence

| **Sample ID** | **Sequence ID** | **C_T_** | **Mapped reads** | **Mean depth** |
| --- | --- | --- | --- | --- |
| **SW20** | Wastewater_seq1 | 33.6 | 105610 | 99384 |
| **SW04** | Wastewater_seq2 | 39.5 | 2115 | 2049 |
| **SW22** | Wastewater_seq3 | 36.5 | 6375 | 6083 |
| **SW24** | Wastewater_seq4 | 37.2 | 82094 | 77388 |
| **SW26** | Wastewater_seq5 | 38.3 | 217785 | 211269 |
| **SW32** | Wastewater_seq6 | 37.4 | 256142 | 243621 |
| **SW78** | Wastewater_seq7 | 40.3 | 309055 | 300480 |
| **SW66** | Wastewater_seq8 | 40.6 | 384195 | 372624 |
| **SW96** | Wastewater_seq9 | 36.2 | 70643 | 67775 |
| **SW96** | Wastewater_seq10 | 36.2 | 8450 | 8114 |
| **SW106** | Wastewater_seq11 | 38.8 | 401595 | 389150 |
