# Supplementary material for: Metabarcoding of Hepatitis E virus genotype 3 and Norovirus GII from wastewater samples in England using nanopore sequencing
Source: Food Environ Virol. Author manuscript; Available in PMC 2023 Dec 1. (PMC7615314; doi:10.1007/s12560-023-09569-w)
Supplement: Supplementary file 8 [file EMS190417-supplement-Supplementary_file_8.docx]

Metabarcoding of Hepatitis E virus genotype 3 and Norovirus GII from wastewater samples in England using nanopore sequencing

Samantha Treagus^1,2^, James Lowther^1^, Ben Longdon^2^, William Gaze^3^, Craig Baker-Austin^1^, David Ryder^1^, Frederico M. Batista^1^

*Author for editorial correspondence:*

Samantha Treagus

UK Health Security Agency

Manor Farm Road

Porton Down

Wiltshire

SP4 0JG

United Kingdom

Email: samantha.treagus2@ukhsa.gov.uk

ORCID iD: 0000-0002-1905-9024

**Online Resource 8**

**Table 1** Closest match using Nucleotide BLAST results for the amplicon consensus sequences

| **Sequence** | **BLAST result** | **Nucleotide identity (%)** | **BLAST origin host and country** |
| --- | --- | --- | --- |
| **Wastewater_seq1** | MH504137.1 | 97.7 | Human, UK |
| **Wastewater_seq2** | MH504146.1 | 98.1 | Human UK |
| **Wastewater_seq3** | MH504128.1 | 97.2 | Human, UK |
| **Wastewater_seq4** | MH504137.1 | 97.7 | Human, UK |
| **Wastewater_seq5** | MW355362.1 | 97.7 | Human, France |
| **Wastewater_seq6** | MH504137.1 | 97.7 | Human, UK |
| **Wastewater_seq7** | MH504146.1 | 95.8 | Human, UK |
|  | MT840367.1 | 95.8 | Wild boar, Italy |
| **Wastewater_seq8** | MT362711.1 | 97.2 | Human, The Netherlands |
|  | MW355220.1 | 97.2 | Human, France |
| **Wastewater_seq9** | MF444141.1 | 98.1 | Human, France |
|  | MF444109.1 | 98.1 | Human, France |
|  | MW355403.1 | 98.1 | Human, France |
|  | MT840367.1 | 98.1 | Wild boar, Italy |
| **Wastewater_seq10** | MF444030.1 | 98.6 | Human, France |
| **Wastewater_seq11** | MH504146.1 | 95.8 | Human, UK |
|  | MT840367.1 | 95.8 | Wild boar, Italy |
