# Supplementary figures and images for: Metabarcoding of Hepatitis E virus genotype 3 and Norovirus GII from wastewater samples in England using nanopore sequencing
Source: Food Environ Virol. Author manuscript; Available in PMC 2023 Dec 1. (PMC7615314; doi:10.1007/s12560-023-09569-w)

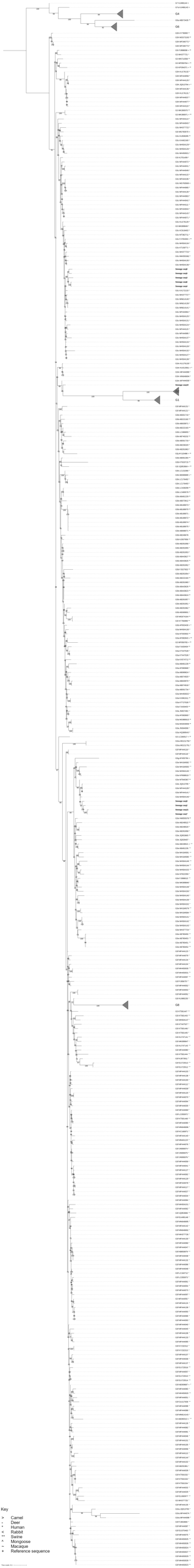

Supplement: Supplementary file 10 [file EMS190417-supplement-Supplementary_file_10.pdf]

Percentage

Sample

Genotype

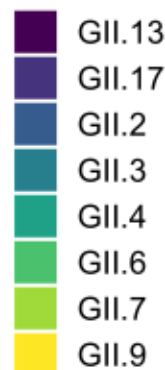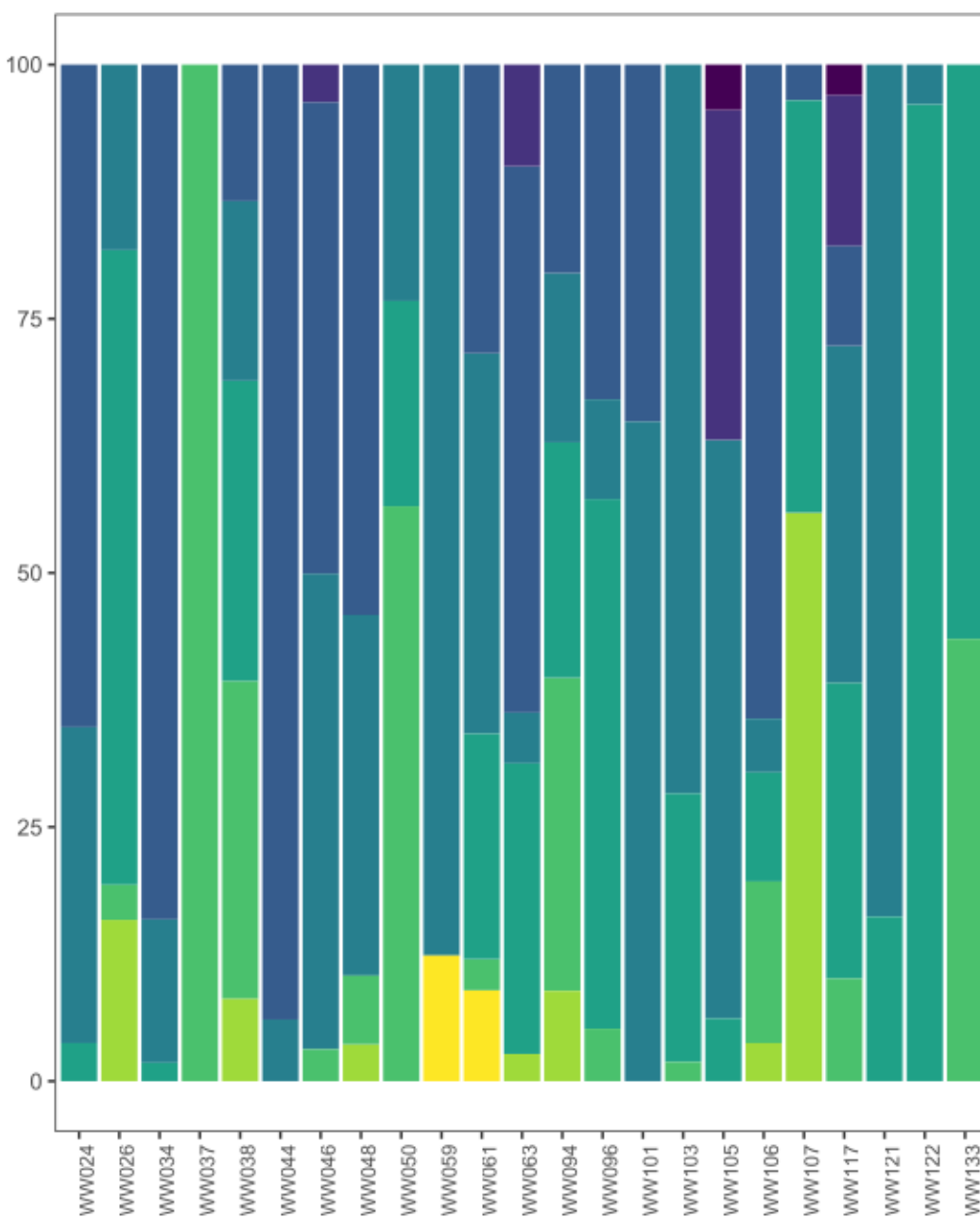

Supplement: Supplementary file 13 [file EMS190417-supplement-Supplementary_file_13.pdf]

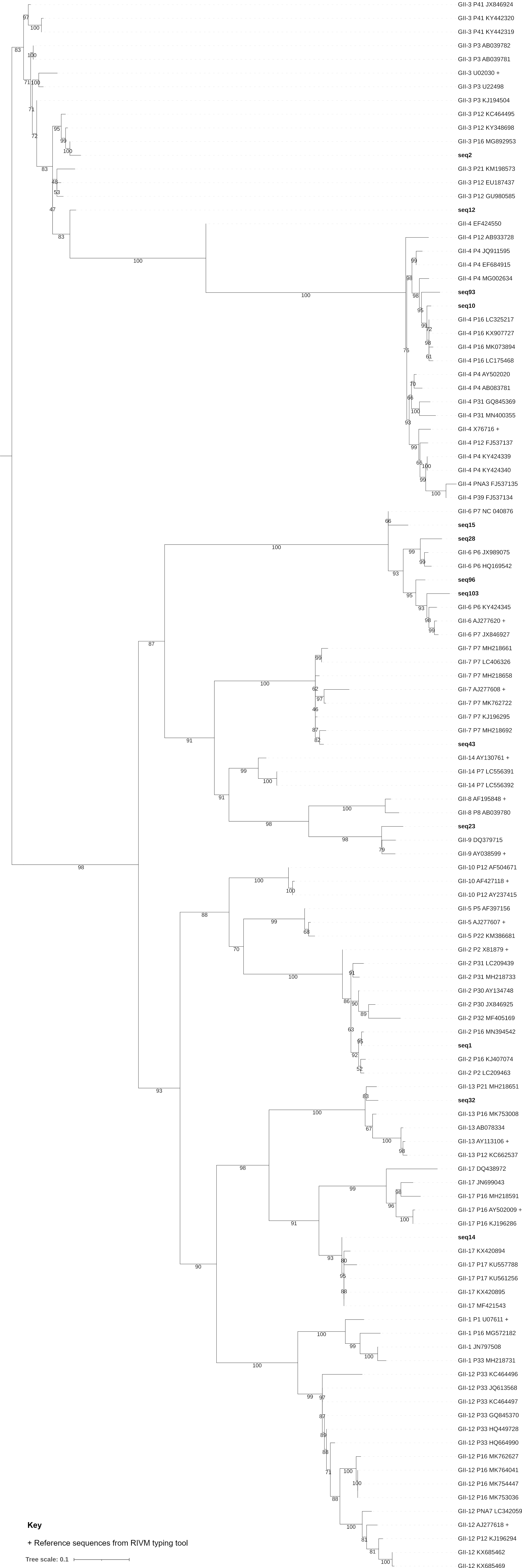

Supplement: Supplementary file 15 [file EMS190417-supplement-Supplementary_file_15.png]
